# The cost of associating with males for Bornean and Sumatran female orangutans: a hidden form of sexual conflict?

**DOI:** 10.1007/s00265-020-02948-4

**Published:** 2020-12-30

**Authors:** Julia A. Kunz, Guilhem J. Duvot, Maria A. van Noordwijk, Erik P. Willems, Manuela Townsend, Neneng Mardianah, Sri Suci Utami Atmoko, Erin R. Vogel, Taufiq Purna Nugraha, Michael Heistermann, Muhammad Agil, Tony Weingrill, Carel P. van Schaik

**Affiliations:** 1 Department of Anthropology, University of Zurich, Zurich, Switzerland; 2 Faculty of Biology and Primates Research Center, Universitas Nasional, Jakarta, Indonesia; 3 Department of Anthropology, Rutgers The State University of New Jersey, New Brunswick, NJ USA; 4 Research Center for Biology, Indonesian Institute of Sciences (LIPI), Cibinong, Indonesia; 5 Faculty of Veterinary Medicine, Bogor Agriculture University, Bogor, Indonesia; 6 Endocrinology Laboratory, German Primate Center, Leibniz-Institute for Primate Research, Göttingen, Germany

**Keywords:** Concealed ovulation, Cost-of-sexual-attraction hypothesis, Fecal cortisol, Socioecology, Sexual coercion, *Pongo* spp.

## Abstract

**Abstract:**

Sexual coercion, in the form of forced copulations, is relatively frequently observed in orangutans and generally attributed to their semi-solitary lifestyle. High ecological costs of association for females may be responsible for this lifestyle and may have prevented the evolution of morphological fertility indicators (e.g., sexual swellings), which would attract (male) associates. Therefore, sexual conflict may arise not only about mating per se but also about associations, because males may benefit from associations with females to monitor their reproductive state and attempt to monopolize their sexual activities. Here, we evaluate association patterns and costs for females when associating with both males and females of two different orangutan species at two study sites: Suaq, Sumatra (*Pongo abelii*), and Tuanan, Borneo (*Pongo pygmaeus wurmbii*). Female association frequency with both males and females was higher in the Sumatran population, living in more productive habitat. Accordingly, we found that the cost of association, in terms of reduced feeding to moving ratio and increased time being active, is higher in the less sociable Bornean population. Males generally initiated and maintained such costly associations with females, and prolonged associations with males led to increased female fecal cortisol metabolite (FCM) levels at Tuanan, the Bornean population. We conclude that male-maintained associations are an expression of sexual conflict in orangutans, at least at Tuanan. For females, this cost of association may be responsible for the lack of sexual signaling, while needing to confuse paternity.

**Significance statement:**

Socioecological theory predicts a trade-off between the benefits of sociality and the ecological costs of increased feeding competition. Orangutans’ semi-solitary lifestyle has been attributed to the combination of high association costs and low predation risk. Previous work revealed a positive correlation between association frequencies and habitat productivity, but did not measure the costs of association. In this comparative study, we show that females likely incur costs from involuntary, male-maintained associations, especially when they last for several days and particularly in the population characterized by lower association frequencies. Association maintenance therefore qualifies as another expression of sexual conflict in orangutans, and especially prolonged, male-maintained associations may qualify as an indirect form of sexual coercion.

**Supplementary Information:**

The online version contains supplementary material available at 10.1007/s00265-020-02948-4.

## Introduction

In most mammals, female reproductive success is limited by access to food resources, while that of males is mainly limited by access to females (Darwin 1871; Emlen and Oring 1977). Hence, males and females have different behavioral strategies to optimize their lifetime fitness, which may lead to sexual conflict (Trivers 1972; Parker 1979). The high male-biased operational sex ratios in species with long lactational infertility and no paternal care may exacerbate sexual conflict (Clutton-Brock and Parker 1992; van Schaik 2016). Orangutan (*Pongo* spp.) females exhibit the longest inter-birth intervals in primates of 6 to 9 years (van Noordwijk et al. 2018), males do not provide direct paternal care for infants (Rijksen 1978; Utami Atmoko et al. 2009), and males are not territorial (Spillmann et al. 2017). Hence, male-male competition for receptive females is high, which carries a high potential for sexual conflict (Trivers 1972; Parker 1979). However, the relative importance of male-male competition, female choice, and sexual conflict in orangutans remains incompletely understood (Nadler 1981; Fox 1998, 2002; Knott 2009; Utami Atmoko et al. 2009; Knott et al. 2010; Spillmann et al. 2010, 2017). There is evidence, however, for the behavioral expression of sexual conflict, in the form of frequent forced copulations (Galdikas 1985a; Mitani 1985; Schürmann and van Hooff 1986; Knott et al. 2010). Females are vulnerable to this form of sexual coercion because of the pronounced sexual dimorphism (Smuts and Smuts 1993), the semi-solitary lifestyle (Rijksen 1978; van Schaik 1999), and the absence of morphological fertility advertisements, such as sexual swellings (Nunn 1999; Zinner et al. 2004). Interestingly, apparent physical injuries resulting from forced copulations have not been reported, and males seem to use only as much force as is necessary to achieve intromission (Knott 2009).

### Sexual conflict over associations

Sexual conflict may arise not only about actual mating but also about association maintenance. Female orangutans are at the solitary end of the fission-fusion spectrum (i.e., females spend on average 50–80% of their time with only their own dependent offspring; van Schaik 1999; van Noordwijk et al. 2009). The low association frequency suggests that the costs of association, in terms of increased scramble feeding competition and hence reduced energy acquisition, are substantial for both males and females (Galdikas 1988; van Schaik and Fox 1996; Utami Atmoko et al. 1997). Yet, associations occur nevertheless, even if only one partner benefits. Specifically, because of the rare siring opportunities, male association decisions may be less cost-sensitive (van Schaik 1999), and males may accept foraging costs due to increased copulation opportunities (orangutans: Mitani 1989; chimpanzees [*Pan troglodytes*]: Emery Thompson and Georgiev 2014; Georgiev et al. 2014). Therefore, males likely benefit from associations with females, as this presumably facilitates monitoring their reproductive status and sexual activities and may be attempts to mate guard females, even if females are often unlikely to be fertile. As a result, females and males may experience a conflict about associating with each other. In fact, females may attempt to reduce time spent in associations because of potential foraging costs (e.g., Knott et al. 2018), whereas males often attempt to prevent them from leaving (van Noordwijk and van Schaik 2009). If this is indeed the case, male-female associations in orangutans can be considered an expression of sexual conflict, and male-maintained associations could be seen as an indirect form of sexual coercion (cf. Muller et al. 2009).

### Absence of fertility advertisement and the cost-of-sexual-attraction hypothesis

Female orangutans do not exhibit any apparent graded, morphological signals advertising fertility (e.g., sexual swellings; Nunn 1999; Zinner et al. 2004). Although rare observations of female proceptive copulations with dominant males have been linked to the peri-ovulatory period (Fox 1998; Knott et al. 2010), ovulation appears largely concealed, as males initiate copulations independent of the females’ reproductive state and during periods of lactational infertility (Nadler 1981; Knott et al. 2010; Kunz 2020). Unpredictable ovulation in other catarrhine primates has been linked to the need to counteract male monopolization and serves to confuse paternity and so offset the risk of infanticide (Hrdy 1979; van Noordwijk and van Schaik 2000; van Schaik et al. 2000, 2004). However, signaling fertility bears costs for females (Matsumoto-Oda 1998; Archie et al. 2014). Particularly, the energetic costs of grouping may have prevented females from evolving signals that advertise prolonged fertility and attract male associates (Slater et al. 2008; Emery Thompson et al. 2014), thereby achieving such paternity confusion. Accordingly, Wrangham (2002) developed the “cost-of-sexual-attraction” hypothesis to explain the variation in morphological fertility advertisement in the genus *Pan*. Indeed, the cost of association for females (i) is negatively correlated with the number of swelling cycles to conception (Deschner et al. 2004; Emery Thompson 2005; Douglas et al. 2016) and (ii) is positively correlated with the rate of sexual coercion and infanticide (Wilson et al. 2014). In a high-quality habitat, both the immediate and delayed benefits to associate with males and to signal fertility over an extended period may outweigh the costs for females. Bonobos (*Pan paniscus*) are at this low cost of association end (Furuichi 2011; Clay et al. 2016; Nurmi et al. 2018) and have very prolonged periods of sexual attractivity (Douglas et al. 2016), and sexual coercion by males is virtually absent (male aggression: Hohmann and Fruth 2003; Paoli 2009; infanticide: Hohmann et al. 2019). The cost of association for chimpanzee females varies geographically. In a more gregarious West African chimpanzee population (*P. troglodytes verus*; Boesch and Boesch-Achermann 2000; Riedel et al. 2011), rates of sexual coercion are low (Stumpf and Boesch 2010) and females may even directly profit from signaling prolonged sexual attractiveness (“Social Passport Hypothesis”; Deschner and Boesch 2007). In contrast, in an East African chimpanzee population (*P. troglodytes schweinfurthii)*, females’ foraging effort is compromised and their energy balance decreases with an increasing number of males in association (Emery Thompson et al. 2014). Consistent with the cost-of-sexual-attraction hypothesis, East African chimpanzees have fewer swelling cycles per conception (Emery Thompson 2005; Deschner et al. 2004; but see Deschner and Boesch 2007), more male sexual coercion (Muller et al. 2007, 2011), and more infanticide (Wilson et al. 2014) than West African chimpanzees.

Following the idea of the cost-of-sexual-attraction hypothesis, the absence of morphological fertility advertisement in the genus *Pongo* may reflect the prohibitively high costs of association to repeatedly signal fertility, while still needing to avoid male monopolization and thus confuse paternity. Here, we evaluate the costs of association in orangutans; future studies will investigate how those relate to the frequency of sexual coercion.

### Geographic variation in sociability in orangutans

In the absence of high predation pressure due to their arboreal life style (van Schaik and van Hooff 1983), food abundance is the major constraint to population density and sociality in orangutans (van Schaik 1999; Hardus et al. 2013; Vogel et al. 2015). Fruit availability is not only thought to be responsible for the higher association frequency in West Sumatran (*Pongo abelii*) (average daily party size ranging from 1.6 to 1.9 individuals) compared to both Eastern Sumatran (*P. abelii*) as well as Bornean orangutans (*Pongo pygmaeus*) (average daily party size ranging from 1.05 to 1.3 individuals) (van Schaik 1999; Mitra Setia et al. 2009; Wich et al. 2011; Roth et al. 2020), it also likely constrains associations within populations over time (van Schaik and Fox 1996; Fox 1998; Wich et al. 2006; Roth et al. 2020; J.Meric de Bellefon et al., unpublished data). A high degree of scramble competition has been held responsible for the low female sociability, and direct female-female contest competition has also been reported (Utami Atmoko et al. 1997; Knott et al. 2008; van Noordwijk et al. 2012; Marzec et al. 2016). The ecological effect on association frequency, however, would be expected to be most prominent in associations between males and females because of the sex differences in ranging patterns and activity budgets connected to their distinct energetic demands (Morrogh-Bernard et al. 2009; van Schaik et al. 2009; Harrison et al. 2010; Vogel et al. 2017). In previous studies, it has been shown that day journey length increases with increasing association size, indicative of increased scramble competition both in Sumatran and Bornean populations (Fox 1998; van Schaik 1999; Wartmann et al. 2010).

In some respects, it appears that the orangutan species have adapted to their distinct conditions, and even under similar food availability in captivity they show a different response to increased sociability. In zoos, Bornean orangutans permanently housed with up to 5 adults exhibited overall higher fecal cortisol metabolite (FCM) levels than the more gregarious Sumatran orangutans living in groups with up to 8 adults, which was attributed to species differences in the sensitivity to social stress (Weingrill et al. 2011). Moreover, captive Bornean orangutans that were kept in fission-fusion like housing systems exhibited lower FCM levels than those kept in a stable group (Amrein et al. 2014). Taken together, these results indicate that social factors, especially extended sociality, lead to a stronger physiological stress response in the less sociable Bornean orangutans, suggesting that they will also prefer lower association rates in the wild.

### Male bimaturism and sexual conflict

Orangutans exhibit a uniquely pronounced male bimaturism, which has been associated with alternative male reproductive strategies (MacKinnon 1974; Utami Atmoko and van Hooff 2004; Pradhan et al. 2012; Dunkel et al. 2013). Unflanged males, who lack secondary sexual characteristics, reportedly associate, copulate, and coerce copulations more frequently than flanged males in the majority of study populations (MacKinnon 1974; Galdikas 1985b; Sugardjito et al. 1987; Knott 2009; Mitra Setia et al. 2009; Utami Atmoko et al. 2009; JAK et al., unpubl. data). Flanged males, who have fully developed secondary sexual characteristics, emit long calls and are reported to rely largely on female choice around conception (Fox 2002; Mitra Setia and van Schaik 2007; Spillmann et al. 2010). Although there is evidence for variation among populations and species in the reproductive strategies of the male morphs (Delgado and van Schaik 2000; Knott and Kahlenberg 2007; Mitra Setia and van Schaik 2007; Utami Atmoko et al. 2009; Spillmann et al. 2017), sexual conflict over associations is likely more pronounced with unflanged males than with flanged males, because the former associate more frequently with females and cannot rely on female choice.

## Aim of the study

Associations and their maintenance may present another, more subtle, context of sexual conflict in addition to forced copulations in orangutans. Following the “cost-of-sexual-attraction” hypothesis (Wrangham 2002), high costs of association may be responsible for the absence of morphological fertility advertisements in female orangutans. Here, we evaluate the costs of association for female orangutans with both females and males at two study sites, Suaq (*P. abelii*, Sumatra) and Tuanan (*P. pygmaeus*, Borneo) using behavioral and endocrine data. Because of large within-species variation in terms of their socioecology (e.g., Vogel et al. 2015; Roth et al. 2020) and little evidence for life history differences between species (van Noordwijk et al. 2018), we refer to study site rather than species differences, as we evaluate only one study site per species. We included female-female associations as a comparative category with the assumption that females have similar incentives to associate with each other (van Noordwijk et al. 2012) as opposed to male-female associations and that these therefore are likely cost-sensitive (sensu van Schaik 1999). We measured behavioral changes (i.e., changes in the daily activity budget) and variation in fecal cortisol metabolite (FCM) levels of parous females in relation to different types of association and social interactions. We hypothesized that (1) if males benefit from monitoring a female’s reproductive state and potentially attempt to monopolize a female’s sexual activities, they likely initiate and maintain such associations with females; (2) social interactions between males and females are rare and therefore females likely do not gain direct social benefits from associations with males (for an exception see Marzec et al. 2016), whereas associations with other females may provide social benefits for their infants (e.g., play opportunities; van Noordwijk et al. 2012); (3) associations with both males and females lead to higher foraging costs, i.e., increased moving and reduced feeding time, for females of the less sociable population, Tuanan, than females of the more sociable population, Suaq; and (4) besides scramble competition, costs of grouping females may bear additional costs from agonistic interactions, especially the occurrence of forced copulations, during associations with males. In Table 1, we provide a detailed overview of hypotheses and predictions.

**Table 1:** Overview of the two main hypotheses evaluated in this study with the corresponding predictions

**Hypotheses and predictions**		**Additional variables**		**Tested in**	
		**Site**†	**Male morph**^‡^	**Tab.**^$^	**Fig.**^$^
**Main hypothesis:** Associations are a context of sexual conflict in orangutans and may therefore qualify as a form of indirect sexual coercion.		T > S	UFM > FLM		
**Part 1:** Male direct benefits to associate with females exceeds those of females					
Association and social interactions (benefits)	Prediction 1.1: Male-female associations are more frequent than female-female associations.^E^	T > S^E^	UFM > FLM^E^	S 1	S 1
	Prediction 1.2: Associations are male-initiated and male-maintained.^E^	T > S^NE^	UFM > FLM^NE^	S 2	1–2
	Prediction 1.3: Male-female associations last longer than female-female associations, indicating that males benefit from prolonged associations.^E Tuanan^	T > S^E^	UFM > FLM^NE^	3	3
	Prediction 1.4: Affiliative social interactions are rare during male-female associations, indicating near-absence of direct social benefits to females from associations with males.^E^	T < S^E^	UFM > FLM^E^ Building up relationship?	S 5	S 2
	Prediction 1.5: Agonistic social interactions outside of the sexual context are rare during male-female associations, indicating near-absence of direct coercive mate guarding by males.^E^	NA	UFM > FLM^O^	S 6	S 2
	Prediction 1.6: Sexual interactions (particularly forced copulations) and genital investigations by males are frequently observed in associations with females, indicating reproductive benefits for males from associations.^E^	T < S^E,O^	UFM > FLM^E^	S 7–8	S 3
**Part 2:** Females incur costs from associations with males as a result of both increased feeding competition and the social interactions meanwhile.					
Cost of association to females	Prediction 2.1: Females’ activity budget changes reflect increased scramble competition when in association with males. Active time increases and the F:M ratio decreases when in association with males.^E^	T > S^E^	Not tested (but P1.1: UFM > FLM^E^)	4 S 9–12	4 S 4
	*Prediction 2.1.1:* Females’ activity budget changes reflect increased scramble competition when in association with any adult individual: Active time increases and the F:M ratio decreases when in association with females.^E^	T > S^E^	NA	4 S 9–12	4 S 4
	Prediction 2.2: Aggression by males imposes further costs on females. Female F:M ratio decreases on days when they experience male aggression (either sexual or non-sexual aggression).^NE (E copulation occurrence at Tuanan)^	T = S^NE^	Not tested (UFM > FLM)	4 S 9–12	5 S 5
	*Prediction 2.2.1:* Aggression by other females also imposes costs on females and therefore, female F:M ratio decreases also on days when they experience aggression from other females, indicating that social stressors affect females’ activity budget.^NE^	T = S^NE^	NA	4 S 9–12	NA
	Prediction 2.3: Female FCM levels are higher on days when in association with males than on days when females are alone with their dependent offspring or in association with other females. Male associates therefore are a social stressor to females.^NE^	T = S^Unk^	Not tested (but P1.1)	5	NA
	*Prediction 2.3.1:* Female FCM levels are also higher on days when females are in association with other adult females than when alone with their dependent offspring. Any adult association partner may qualify as a social stressor to females.^NE^	T = S^Unk^	NA	5	NA
	Prediction 2.4: Female FCM levels increase with an increasing number of days spent in association with any association partner as a result of the accumulating negative energy balance, indicating energetic stress.^E^	T > S^Unk^	NA	5	6
	Prediction 2.5: Female FCM levels are higher on days when they experience forced copulations, indicating either social (Soc.) or energetic (Eco.) costs.^NE^	Soc. T = S Eco. T > S	Not tested (UFM > FLM)	5	7
**Main hypothesis 2:** Female orangutans do not exhibit any apparent morphological fertility advertisements, because of the prohibitively high costs of association (cost-of-sexual-attraction hypothesis prediction 1), while needing to confuse paternity.					
	Prediction 3.1: Females incur costs from associations with males, which are consistent with scramble competition of grouping (predictions 2.1, 2.4).^E^	T > S ^E^	NA	4, 5	4, 6
	Prediction 3.2: The frequency of male-female associations increases with the age of the dependent offspring indicating some reproductive benefits (conception and paternity manipulation), while female-female association remains constant over the age of the dependent offspring (indicating socializing benefits).^E^	NA	NA	S 1	S 1

Predictions include the evaluation of social benefits (P1.1–1.6) and costs resulting from both associations and social interactions (P2.1–2.5) during associations between males and females. Observed cost-benefit balances and thus, predictions may vary with both study site (^†^) and male morph (^‡^). The two right columns indicate where the corresponding test (table with model output and figure) can be found

^†^Association frequency is reportedly higher at Suaq (S) than at Tuanan (T), indicating generally lower costs of association at Suaq than at Tuanan. Accordingly, each prediction may vary with the socioecological background of the study site (“T > S”: prediction is more pronounced at Tuanan than Suaq; “T < S”: prediction more pronounced at Suaq than at Tuanan; “T = S”: no site difference expected)

^‡^The two male morphs follow different reproductive tactics. While flanged males (FLM) are reportedly preferred by females, associate, and copulate more selectively, unflanged males (UFM) associate, copulate, and coerce more frequently at Suaq and Tuanan (Kunz 2020). Both the extent of social benefit and the cost of association inflicted on females may therefore vary with male morph and their reproductive tactic

^$^The “S” in front of the number indicates that the table or figure, respectively, can be found in the supplementary materials

*E*, evidence for this prediction was found in the current study; *NE*, no evidence was found for this prediction in the current study; *Unk*, not enough evidence to either support or disapprove the prediction, often because of limited data sets; *O*, evidence for the opposite pattern (for study site and male morph comparison)

## Methods

### Study sites and study subjects

Behavioral focal data on individually recognized adult females were collected at the long-term field sites of Tuanan, Mawas Reserve, Central Kalimantan, Indonesia (02° 15′ S; 114° 44′ E) and Suaq, Gunung Leuser National Park, South Aceh, Indonesia (03° 02′ N; 97° 25′ E) between July 2003–July 2018 and June 2007–March 2018, respectively. Because parous females are in continued association with their dependent offspring (van Noordwijk et al. 2009) and lactate over multiple years (van Noordwijk et al. 2013), both association patterns and the cost-benefit balances incurred by sociability are likely different from nulliparous (adolescent) females (van Schaik et al. 2009; Ashbury et al. 2020). Therefore, only data on parous females with a dependent offspring were included in this study (*N* = 20 females [Suaq: 6; Tuanan: 14]). Similarly, females who had lost their infants due to unknown reasons (Marzec et al. 2016; MAvN et al. unpubl. data) were excluded from the analyses after the loss of their infants, until they had given birth to a new infant. Infant loss is an extremely rare event (van Noordwijk et al. 2018), and insufficient data were available to add them as a separate category. The age of the dependent offspring of females was taken as a proxy for their reproductive state and included in all the analyses (Table 2). Infant ages were either known because the birth was directly observed or estimated from the first time an infant was observed (Table 2; cf. van Noordwijk et al. 2018).

**Table 2:** Overview of the data available to assess the cost of association for parous females at Tuanan and Suaq

Study site	Name of parous female	Dependent infant		Activity budget		Hormone samples	
		Minimum age of infant (years)	Maximum age of infant (years)	Number of follow periods	Number of full-day focal follows^§^	Total samples available	With behavioral reference*
Suaq	Cissy	1.6	5.2	4	35	42	13
Suaq	Ellie	0.3	2.7	4	37	32	14
Suaq	Friska	0.9	4.9	8	51	33	10
Suaq	Lisa	0.5	7.7	11	77	60	15
Suaq	Raffi	0.8	1.7	1	6	12	0
Suaq	Sarabi	0.6	0.9	2	15	4	0
Tuanan	Cikipos	3.2	3.2	1	5	6	0
Tuanan	Cinta	0.9	2.7	3	17	12	5
Tuanan	Desy	0.0	5.8	13	105	54	37
Tuanan	Inul	0.1	3.3	12	77	44	24
Tuanan	Jinak	0.3	7.1	45	365	66	39
Tuanan	Juni	0.0	6.6	33	255	88	51
Tuanan	Kerry	0.0	7.5	40	280	65	38
Tuanan	Kondor	0.0	1.9	10	64	23	12
Tuanan	Milo	0.1	1.1	3	20	9	6
Tuanan	Mindy	0.1	6.9	52	390	95	65
Tuanan	Pinky	0.0	7.5	6	39	25	7
Tuanan	Sidony	0.0	6.1	6	58	45	25
Tuanan	Sumi	0.4	3.4	20	154	0	0
Tuanan	Tina	3.7	5.5	5	36	27	9

*Number of samples included in analyses

^§^Number of days included in the analyses

### Behavioral data

#### Activity budget

Behavioral data were collected according to an established, standardized protocol (https://www.aim.uzh.ch/de/orangutannetwork/sfm.html). We collected 2-min instantaneous data during full-day female focal follows on their activities (feeding, moving, resting, and social interactions). We recorded all occurrences of any individual in association (within 50-m distance) per 2-min interval and ad libitum social interactions with the focal individual. Social partners included the female’s own dependent and independent offspring, adolescent individuals, and adult females and males (unflanged and flanged). We subdivided social interactions into sexual, affiliative, and aggressive interactions. Sexual interactions comprised genital investigations by males, copulations, and copulation attempts. Copulations were labelled as either forced, if the female showed any resistance behavior (e.g., repeated attempt to move away, struggling against the males attempt to intromit), or unforced (following the definition of Fox 1998). We grouped aggression between females and both males and other females (excluding forced copulations) into non-physical (displays, short chases) and physical aggression (fights or coercive hand holding by males (van Schaik et al. 2006)). Affiliative interactions comprised allo-grooming, touching another individual, sitting in body contact, and begging for and sharing food. Because focal animals were individually recognized, we could not collect blinded data. Only data from well-trained observers with high inter-observer reliability were included in the analyses.

#### Association patterns and social interactions

Male focal follows collected with the same methods as the female focal follows were used to enhance the data set on the duration of associations and a fuller record of male-female dyadic interactions resulting in 960 male-female associations (Suaq: 292; Tuanan: 668) with known start and end times. An association between two individuals could last for multiple days and contain breaks, i.e., the association partners were at a distance of more than 50 m. Orangutans most likely perceive the presence of other individuals at distances of more than 50 m better than humans on the ground (van Noordwijk et al. 2009, 2012), and therefore, brief “separations” (> 50-m distance) likely are not relevant to them. If breaks lasted for longer than one full-day focal follow, we considered it as two separate association units. We recorded the individual responsible for any distance changes (in distance classes: contact, no contact < 2 m, 2–5 m, 5–10 m, 10–50 m) during the association, as well as the initiator (first approach to < 50 m) and terminator (who left to > 50 m) of associations. We calculated the Female Hinde Index (FHI) for female-male associations based on these approaches and leaves as follows:

$$ \mathrm{Female}\ \mathrm{Hinde}\ \mathrm{Index}\ \left(\mathrm{FHI}\right)=\left(\frac{\mathrm{approaches}\ \mathrm{by}\ \mathrm{female}}{\mathrm{approaches}\ \mathrm{by}\ \mathrm{female}+\mathrm{approaches}\ \mathrm{by}\ \mathrm{male}}\right)-\left(\frac{\mathrm{leaves}\ \mathrm{by}\ \mathrm{female}}{\mathrm{leaves}\ \mathrm{by}\ \mathrm{female}+\mathrm{leaves}\ \mathrm{by}\ \mathrm{male}}\right) $$

A positive FHI indicates that the female was on average responsible for the maintenance of the association, while a negative FHI stands for a male-maintained association (Hinde and Atkinson 1970). The FHIs were calculated over all known approach and leave events and distance classes per association. Detailed approach and leave data throughout the association for the FHIs is available for 665 male-female associations (Suaq: 223; Tuanan: 442).

### Ecological data

The monthly Fruit Availability Index (FAI; percentage of trees with fruits over all surveyed trees) was obtained from monthly phenology surveys of ~ 1500 trees at Tuanan and ~ 1000 trees at Suaq (Harrison et al. 2010; Vogel et al. 2015) (Table 3). Because the FAI is generally higher at Suaq than at Tuanan (Wich et al. 2011), we *z*-transformed all the FAIs within study site prior to the analyses (zFAI) to assess local FAI effects rather than between site comparisons. Although the FAI does not include fruits from lianas, which are components of orangutan diet, it corresponds well to the total proportion of fruits, i.e., high-quality food items, in their diet (Vogel et al. 2017), and can thus be taken as a proxy for forest productivity (Vogel et al. 2015).

**Table 3:** Definition of fixed factors included in the activity budget analyses based on female full-day focal follows (*z*, continuous variables were *z*-transformed prior to analyses)

Type	Factor	Definition
Social	z Cumulative male association hours	The sum of all association time spent with males during a full-day focal follow, e.g., if a female was 1 h in association with male A and 5 h with male B on a full-day focal follow, it would result in 6 cumulative male association hours^$^
	z Cumulative female association hours	The sum of all association time spent with females during a full-day focal follow^$^
	Number of consecutive days with males	The number of (known) days a female focal animal was in association with males, independent of the males’ identity, e.g., the female may have been in association with male A for 2 days and with male B the next day, which would be 3 consecutive days with males
	Number of consecutive days with females	The number of (known) days a female focal animal was in association with females, independent of the female partner’s identity
	Number of copulations	Number of observed copulations during the female full-day focal follow
	Male-female cumulative aggression index	We combined the occurrence of forced copulations and other male aggression to a daily male-female cumulative aggression index, coded for severity (0 = no aggression; 1 = aggression not directly in a sexual context and no physical contact, such as displays, chases and displacements; 2 = forced sexual interactions)
	Female-female agonistic interactions	Days with female-female aggression were rare (*N* = 2 [Suaq], 16 [Tuanan]), and could only be included as presence/absence data, and not coded for the severity
	Social interaction time (h)	The total time spent in social interactions with any partner (including own dependent infant and association partners) during a full-day focal follow (social interactions account for ~ 0.5% of the total active time [Tuanan: 0.4%; Suaq: 1.1%])
	Site (Suaq vs. Tuanan)	Population differences may be the result of either species or ecological differences between the two study populations. Forest productivity is generally higher at Suaq (Sumatra, *P. abelii*) than at Tuanan (Borneo, *P. pygmaeus wurmbii*) (Wich et al. 2011).
Ecological	z Fruit Availability Index	Monthly percentage of trees with fruits over all surveyed trees based on monthly surveys (~ 1500 trees at Tuanan and ~ 1000 trees at Suaq)
Physiological	z Age of dependent offspring (years)	Infant ages were either known because the birth was directly observed, or estimated from the first time an infant was observed (Table 2)

^$^We chose to include the daily cumulative hours spent with either adult females or males to account for multiple individuals in association and the duration spent with each of them (including both association time and the number of individuals as separate variables would have led to multi-collinearity issues)

### Endocrine data

#### Collection, preservation, and extraction of fecal samples

We measured fecal cortisol metabolite (FCM) levels for females during association and non-association days. Fecal material was collected non-invasively, when individuals defecated naturally. Because there is an excretion lag time of 24–72 h for fecal cortisol metabolites (Weingrill et al. 2011), samples were collected on at least 5 consecutive days, once a day, preferably in the morning. Due to individual ranging patterns in orangutans, samples could only be taken during focal follows lasting 5–10 days with at least 5 weeks between successive sample periods, because individuals were not followed during this time. The methods to preserve and extract fecal samples from orangutans for hormone analyses have been established and validated (Weingrill et al. 2011; Amrein et al. 2014; Marty et al. 2015; Nugraha et al. 2016). Because of logistic constraints and varying infrastructures at the two field sites, different preservation and extraction methods had to be used. Generally, the fresh feces were homogenized using a stick and a 2–5-g aliquot was collected for analysis. Only samples not contaminated with urine were taken. When electricity from solar power was available, the fresh feces were collected into a polypropylene tube and frozen at − 18 °C upon return to the field station in the evening. All samples remained frozen until transported to the endocrinology laboratory at Bogor Agricultural University where samples were lyophilized, pulverized and subsequently extracted with 80% methanol in water as described in detail elsewhere (Weingrill et al. 2011; Nugraha et al. 2016). At Suaq and when electricity supply was not guaranteed at Tuanan, fecal samples were placed in a tube containing 5 ml of 80% ethanol in water for preservation upon collection. Samples were extracted upon return to the field station using a field-friendly, previously validated extraction method (Nugraha et al. 2016). Although these extraction methods have been shown to produce results which are strongly correlated (Nugraha et al. 2016), we controlled for potential extraction method differences by normalizing all FCM measurements within individual and method using *z*-transformations in the statistical analyses (van de Pol and Wright 2009; for details see data analysis section).

#### Hormone measurement

Fecal cortisol metabolite levels were measured in a total of 745 samples (Table 2) using a microtiter plate enzyme immunoassay (EIA) for 11ß-hydroxyetiocholanolone (Ganswindt et al. 2003), a major metabolite of cortisol in primate feces (Heistermann et al. 2006). The assay has been previously validated and successfully applied for assessing adrenocortical activity in numerous primate species (e.g., Heistermann et al. 2006) including captive and wild orangutans (Weingrill et al. 2011; Amrein et al. 2014; Marty et al. 2015). Samples used for this study were analyzed in different cohorts at two different laboratories (German Primate Center, DPZ, by A. Heistermann and Bogor Agricultural University, IPB, by JAK), with the locality of analysis for each sample included as a fixed effect in the statistical analyses (results remain the same if we standardize by both laboratory, method and individual, and are not shown below). EIAs were performed as previously described (Heistermann et al. 2004). Samples from the same individual were analyzed on the same microtiter plate, whenever possible. Each sample was analyzed in duplicate. We remeasured samples with a coefficient of variation (CV) > 7% between duplicates. Moreover, we reran any microtiter plate for which the intra-assay CV of the internal high- and low-value quality controls exceeded 10%. For the samples analyzed at both IPB and DPZ, the intra-assay CVs were below 10%, and the inter-assay CVs did not exceed 15%. All FCM concentrations are expressed in ng/g dry fecal weight.

### Statistical analyses

All the statistical analyses were conducted in R version 3.5.2 (R Core Team 2018). We ran (generalized) linear mixed effect models ([G]LMM) using the “lme4” and “lmerTest” packages (Bates et al. 2015; Kuznetsova et al. 2017). Model assumptions (normality [for LMMs], homoscedasticity) were checked by the visual inspection of residual plots. Variance Inflation Factors (VIF) were calculated to examine potential multi-collinearity issues using the “car” package (VIF < 2, for the full model without interaction terms included and VIF < 4 for the full model with interaction terms) (Fox and Weisberg 2018). Further, we checked all the models for influential cases and outliers (Cook’s distance from the package “influence.ME” by Nieuwenhuis et al. 2012). The *P* value of 0.05 was used as a cutoff value for significance. For all statistical analyses, full models including all variables (social, ecological, and physiological factors) and their possible 2nd-order interactions (if applicable) were set up, and compared to the control model, containing all the random and control (ecological and physiological) factors, using likelihood ratio tests. All figures were generated using the “ggplot2” (Wickham 2016) and “cowplot” (Wilke 2019) packages.

#### Behavioral data—association patterns and maintenance

We evaluated the time (average daily hours) females spent in association with either other females or unflanged and flanged males during follow periods in separate analyses (LMM) and with the study site, zFAI, and the age of the dependent offspring as fixed effects. Individual identity was added as a random intercept.

We assessed when associations were male-maintained by setting up a binomial GLMM based on the FHI values (male-maintained when FHI < 0). We added study site, male morph, the age of the dependent offspring of the female (years), local fruit availability (zFAI), the occurrence of copulations (both unforced and forced), and association duration as fixed effects. To account for having the same individuals in several association dyads, both female and male identities were added as crossed random intercepts.

We formulated a Cox proportional hazard mixed model (survival analysis) using the package “coxme” (Therneau 2018) to evaluate if male-female associations lasted over more consecutive days than female-female associations based on the female focal follow data. We used right-censored data to account for unknown association endings, because females were no longer followed despite still being in association (*N* = 625 associations [Suaq: 61 associations with females, 53 with flanged males and 173 with unflanged males; Tuanan: 81 with females, 96 with flanged males, 161 with unflanged males] during 167 female FPs and of 21 females). We included both associations with known and unknown start times, because excluding associations with unknown start times (~ 40% of association dyads) would have introduced a bias against long associations in the analysis (for further details on this issue and for the results excluding associations with unknown start time, see supplementary mat). Besides the type of adult association partners (female, unflanged and flanged male), we added study site, zFAI, and the age of the dependent offspring (years) as fixed effects in the model. We set contrasts for the association partner type to first compare association maintenance between male and female association partners and then the two male morphs. Further, we included the follow period nested in the female identity as a random intercept to avoid pseudo-replication.

#### Behavioral data—activity budget changes

The daily activity budget was calculated from the 2-min instantaneous data taken during full-day female focal follows (*N* = 2086; Suaq: 221; Tuanan: 1865), and thus, (1) it includes the association record over the entire day, and (2) it accounts for activity budget variation over daytime. To account for variation in activity budgets (van Noordwijk et al. 2012), we only included female follow periods (FP) that contained at least 5 full-day focal follows (mean = 8.3 ± SE 0.1) within 40 days (on average within 9 days) in the analyses. First, we evaluated the variability of the total active time, which comprised the total hours from leaving the morning nest to entering the evening nest. Second, we evaluated if female foraging behavior changed on days with associations and social interactions by analyzing variation in daily feeding time while controlling for moving time (offset term) (henceforth referred to as F:M ratio). Daily moving hours correlate strongly with day journey length (DJL) (Pearson correlation for available Tuanan data: *R*^2^ = 0.76, *t*_769_ = 32.66, *P* < 0.0001, *N* = 771 female full-day follows). We analyzed daily moving hours rather than DJL, because it is a proxy for daily travel, but also includes moves within feeding patches, with no net displacement in space, which are likely not accurately reflected in DJL. We tested for the effects of social, physiological and ecological factors on active time and F:M ratio in linear mixed models. We built in the female follow period (FP) nested in female identity as random intercepts to avoid pseudo-replication. The separate analyses on the changes of all activity budget components (feeding, resting, and moving hours) including separate analyses for each study site are reported in the supplementary materials (STable 11–14).

As social factors, we included the total cumulative time spent with either males or females and any agonistic and sexual interaction recorded as fixed effects (details in Table 3). Because consecutive association days are likely inter-dependent and there may be compensatory effects, we also included the total number of (known) consecutive days in association with either males or females in the full model. Further, we controlled for potential confounding physiological and ecological factors, overarching site differences (Tuanan, Suaq), and the total time spent in social interactions with any partner (Table 3). We tested for interaction terms between study site and social factors to check for population differences. Interaction terms were only included in the final model if they improved the model fit based on likelihood ratio tests. Both control models – including study site, zFAI, age of the dependent offspring and total social interaction time – significantly improved the null models, containing only the random intercepts (and the offset term) (active time: *χ*^2^_4,8_ = 51.75, *P* < 0.001; F:M ratio: *χ*^2^_4,8_ = 53.08, *P* < 0.001). We excluded 16 days when females fed less than 1 h and their active time was below 6 h because of serious health issues or lack of habituation, as these days revealed to be influential cases and the model assumptions were violated (for one context of these outliers see Marzec et al. 2016).

#### Endocrine data

The behavioral reference day corresponding to the measured FCM level was obtained by backdating 3 days from the date of collection of morning fecal samples and 2 days for samples collected after 2 pm (Cadilek 2009; Weingrill et al. 2011; Amrein et al. 2014; Nugraha et al. 2016). If there were several fecal samples for one behavioral reference day, only the morning sample was included in the final analysis. FCM levels with the same behavioral reference day were strongly correlated (*r* = 0.73, CI = [0.60, 0.83], *P* < 0.0001, *N* = 78). To control for any sample hour bias, the time of sample collection (i.e., time of defecation) was included in the statistical analyses as a control factor although previous data on captive-housed animals showed no time-of-day effect (Weingrill et al. 2011). FCM levels were ln-transformed to normalize their distribution. Subsequently, the values were standardized within individual and extraction method used, to be able to assess FCM level changes caused by social and ecological stressors within individuals rather than between individuals (method described in van de Pol and Wright 2009). Because such *z*-transformations may be sample size dependent, we only included those individuals in the analyses for which more than 10 samples and at least 5 known behavioral reference days for a given extraction method were available. The within-individual transformations were done including all available samples, including the samples without behavioral reference (*N* = 745). The analysis included only the samples with a known behavioral reference day (*N* = 370). The number of total samples available (per method and individual) was included in the analysis as a control factor. A linear mixed effect model (LMM) was set up to test for the effect of social factors on female FCM levels. The same social factor categories as described in the activity budget analyses were tested. The time to sample extraction (days), the total number of days an individual was followed, the age of the dependent infant (years), an activity budget parameter (feeding proportion), and the Fruit Availability Index (FAI) were included in the full model to control for possible confounding factors leading to FCM changes. The female follow period was added as a random intercept to avoid pseudo-replication. Because the FCM levels were standardized within individual and method, these two factors were not included as random intercepts in the analysis to keep the models as parsimonious as possible. The analyses without the standardization procedure and including individual identity and extraction method as random intercepts yielded the same patterns and are reported in the supplementary materials. The control model with all the potential confounding factors did not improve the model fit of the null model containing the random intercept term only (*χ*^2^_3,12_ = 6.96, *P* = 0.64, ΔAIC = 11.04).

## Results

### Initiation and maintenance of associations

#### Association frequency

Despite substantial day-to-day variation, females spent on average (mean) 30.0 ± SE 0.1 min per day in association with other females (Suaq: 82.6 ± SE 13.6 min; Tuanan: 23.7 ± SE 2.4 min), 53 ± SE 0.1 min with unflanged males (Suaq: 200.1 ± SE 20.0 min; Tuanan: 35.1 ± SE 3.1 min), and 20 ± SE 0.0 min with flanged males (Suaq: 29.1 ± SE 6.8 min; Tuanan: 19.2 ± SE 2.3 min) (SFig. 1). The time females spent in association with both flanged and unflanged males increased as the age of their dependent offspring increased (flanged: *β* = 0.448 ± 0.090, *t* = 5.001, *P* < 0.001; unflanged: *β* = 0.606 ± 0.126, *t* = 4.799, *P* < 0.001; STable 1; SFig. 1), while the association time with other parous females remained constant with offspring age (*β* = 0.067 ± 0.063, *t* = 1.066, *P* = 0.29; STable 1; SFig. 1). Time spent in association with other parous females and unflanged males was generally higher at Suaq than at Tuanan (females: *β* = − 1.058 ± 0.360, *t* = − 2.939, *P* = 0.008; unflanged: *β* = − 1.821 ± 0.556, *t* = − 3.273, *P* = 0.003; STable 1; SFig. 1), but not with flanged males (*β* = 0.228 ± 0.332, *t* = 0.688, *P* = 0.5). To sum up, females were more frequently in association with unflanged males than with adult females or flanged males, partly supporting prediction 1.1 (Table 1), and time in association with both male morphs increased with the age of the dependent offspring of females (Table 1: prediction 3.2).

#### Association initiation and maintenance

**Fig. 1:**
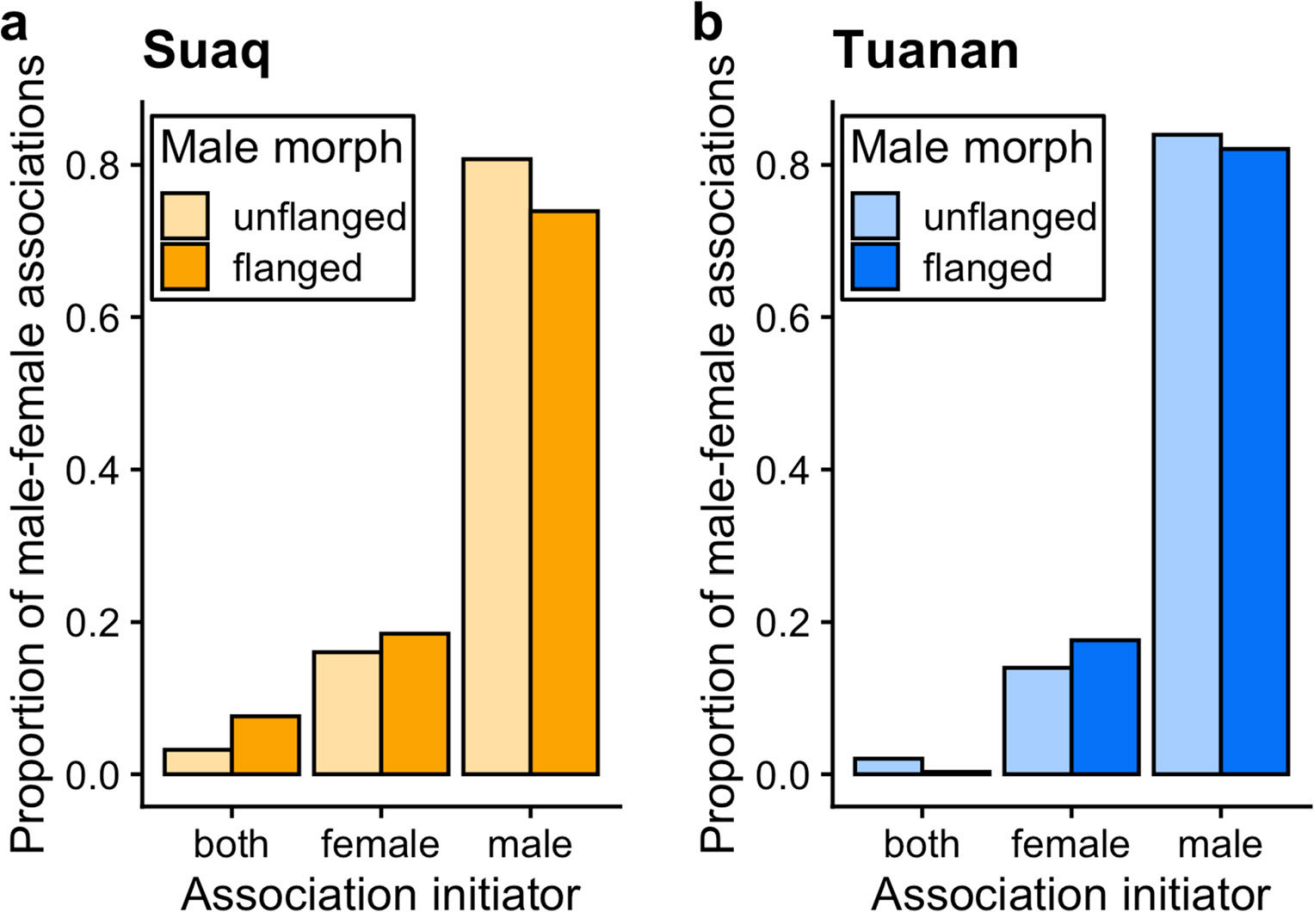
Proportion of association initiations by either both, female or male by study site (**a** Suaq, **b** Tuanan) and male morph. Only associations with a known initiator are included (*N* = 957 [Suaq: 279; Tuanan: 678])

**Fig. 2:**
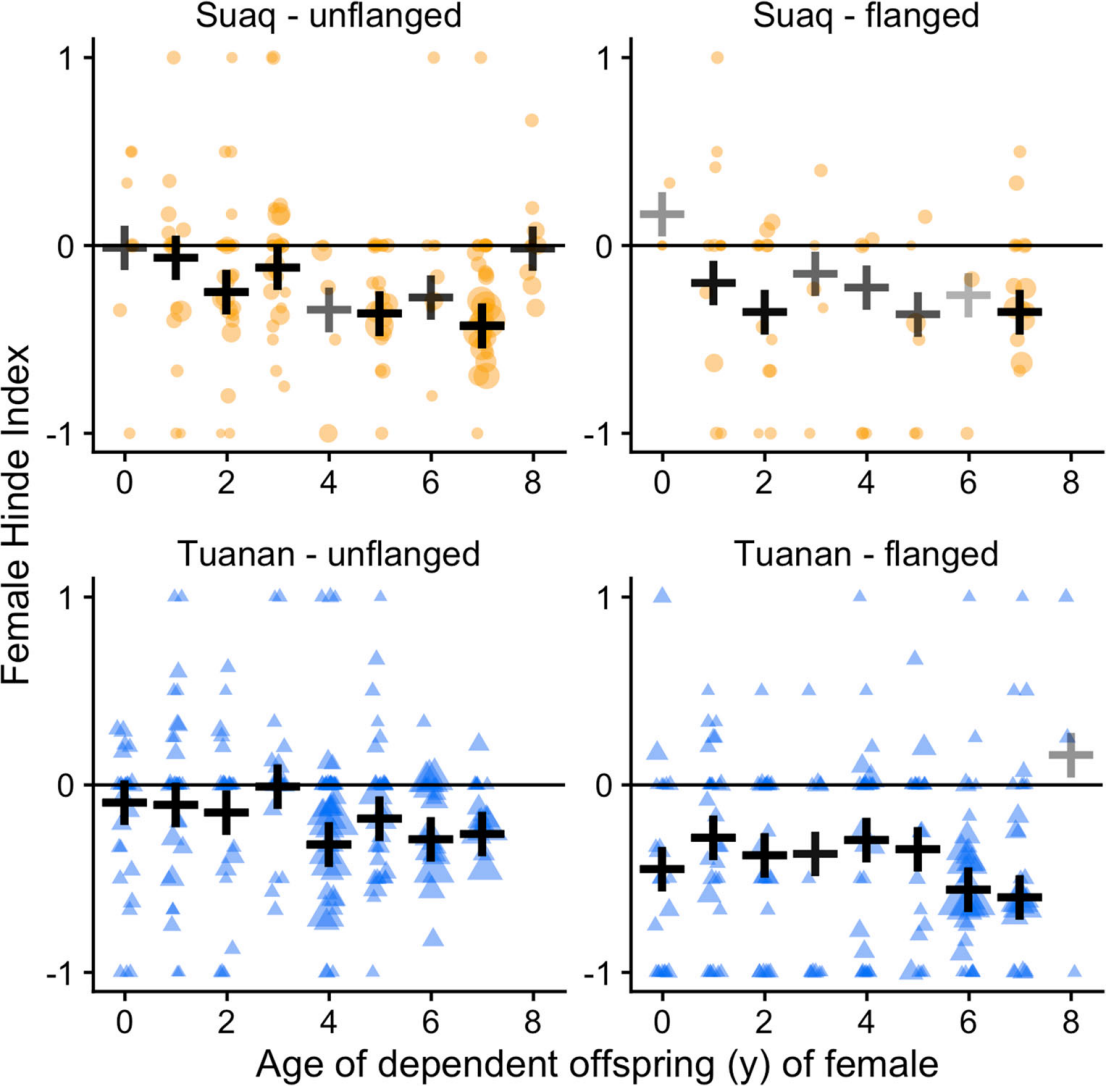
Female Hinde Index of associations with unflanged males (left) and flanged males (right) by study site (Suaq: top; Tuanan: bottom) by the age of the dependent offspring (year), as a proxy for female reproductive status. The black crosses indicate the weighted mean FHI (by the number of known approaches and leaves) and their transparency is relative to the number of associations included. Data points (Suaq: orange; Tuanan: blue) represent individual association units and only include known approaches and leaves (*N* = 665). The data point size is relative to the association duration and a horizontal jitter function was applied to the data points to make overlapping data points more visible

Both flanged males (Tuanan: 82.1%; Suaq: 73.9%) and unflanged males (Tuanan: 84.0%; Suaq: 80.7%) initiated associations with females more frequently than the females themselves (Fig. 1). Moreover, both flanged males (Suaq: mean[FHI] = − 0.25 ± SE 0.06 [*N* = 63 associations]; Tuanan: − 0.38 ± SE 0.03 [*N* = 205]) and unflanged males (Suaq: − 0.16 ± SE 0.03 [*N* = 160]; Tuanan: − 0.12 ± SE 0.03 [*N* = 237]) maintained these associations (Fig. 2). The full model for the probability that associations were male-maintained explained significantly more variability than the null model (*χ*^2^_3,9_ = 72.53, *P* < 0.0001, *N* = 665 of 30 female and 140 male identities; STable 2). First, especially long associations were more likely male-maintained (*β* = 1.226 ± 0.255, OR = 3.40, *z* = 4.804, *P* < 0.001). Second, flanged males were more likely to maintain associations with females than unflanged males (*β* = 0.653 ± 0.201, OR = 1.92, *z* = 3.244, *P* = 0.001), whereas this difference between male morphs was more pronounced at Tuanan than at Suaq, as the model fit marginally improved when adding this interaction term (*χ*^2^_9,10_ = 4.12, *P* = 0.04). Association maintenance by males was independent of the female’s dependent offspring’s age (*β* = 0.089 ± 0.100, OR = 1.09, *z* = 0.891, *P* = 0.37), the local zFAI (*β* = − 0.019 ± 0.089, OR = 0.98, *z* = − 0.215, *P* = 0.83), and the occurrence of sexual interactions (*β* = 0.556 ± 0.298, OR = 1.74, *z* = 1.866, *P* = 0.06). In sum, prediction 1.2 (Table 1) was supported, as associations were male-initiated and male-maintained independent of the female reproductive state, whereas this was the case at both study sites and by both male morphs.

#### Association maintenance over multiple days

**Fig. 3:**
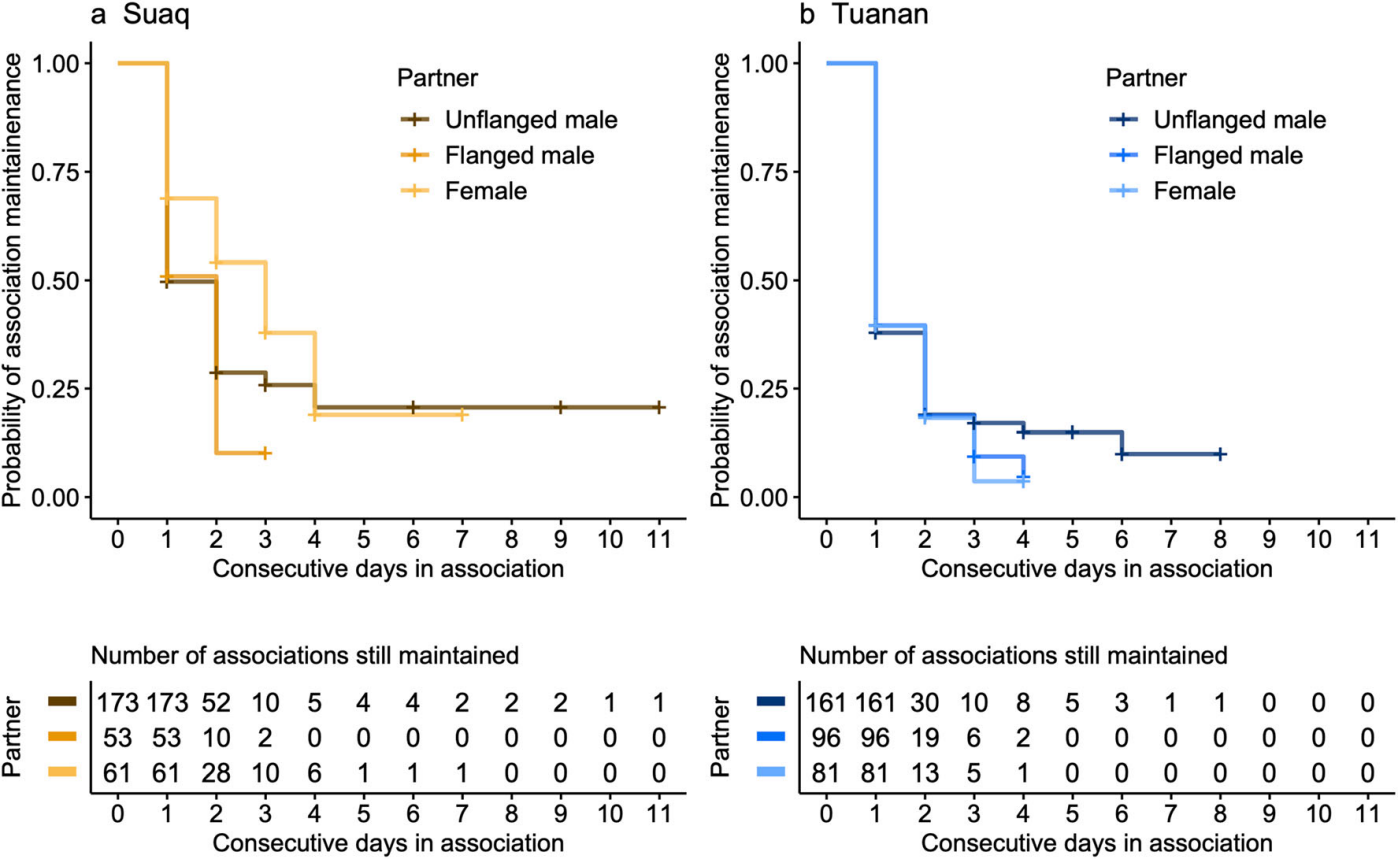
Kaplan-Meier survival curve for the maintenance of associations over consecutive days at Suaq (**a**) and Tuanan (**b**) by the association partner type (color). The survival curve is based on the female focal data from follow periods also including the non-full-day focal follows (e.g., days when an individual was found) (*N* = 625 [Suaq: 287; Tuanan: 338] associations of 21 females and 168 different FPs). The left-censored data is indicated in crosses

Male-female associations were maintained over more consecutive days at Tuanan (maximum 8 days) than female-female associations (maximum 4 days), whereas at Suaq this difference between the maintenance of male-female (maximum 11 days) and that of female-female (maximum 7 days) associations was less pronounced (Fig. 3; Table 4). Accordingly, the survival analysis on the probability of ending an association was significantly better when including the interaction between study site and partner type (*β* = − 0.234 ± 0.089, HR = 0.79, *P* = 0.009): Female-female associations ended sooner at Tuanan than male-female associations; at Suaq this difference was less pronounced (Fig. 3). We could not find any difference in association maintenance probability between the two male morphs (unflanged vs. flanged) (Table 4). Associations were maintained over more consecutive days with the increasing age of the dependent offspring of a female (*β* = − 0.169 ± 0.066, HR = 0.84, *P* = 0.01). The interaction between the age of the dependent infant and partner type did not improve the model fit (*χ*^2^_2_ = 0.83, *P* = 0.66). Local zFAI did not have an effect on the association maintenance (Table 4). All in all, both unflanged and flanged males maintained associations with females over more consecutive days than females did with other females at Tuanan, the less sociable population, whereas we find no such difference at Suaq, the more sociable population, supporting prediction 1.3 (Table 1) and its site difference but not the male morph component.

**Table 4:** Probability of ending an association: output of the Cox proportional hazard mixed model for the total number of (known) days in association by the type of association partner, study site, age of the dependent offspring (years), and zFAI (*χ*^2^_7_ = 28.80, *P* = 0.0002, *N* = 625 associations of which 426 with known end, of 21 female identities and 167 FPs). All fixed effects with *P* < 0.05 are indicated in bold

	*β*	SE	Hazard ratio	*z*	*P*
Site (Suaq vs. Tuanan)	0.551	0.146	1.74	-	-
Association partner					
Sex (male vs. female)	0.233	0.071	1.26	-	-
Male morph (unflanged vs. flanged)	− 0.029	0.103	0.97	-	-
**Age of dependent offspring (years)**	− 0.169	0.066	0.84	**− 2.560**	**0.010**
z Fruit Availability Index	− 0.023	0.063	0.98	− 0.360	0.720
**Site: association partner sex (male vs. female)**	− 0.234	0.089	0.79	**− 2.620**	**0.009**
Site: partner male morph (unflanged vs. flanged)	− 0.019	0.130	0.98	− 0.150	0.880

#### Social interactions between males and females

Affiliative social interactions occurred in 6.0 ± SE 0.8% of all male-female associations (STable 5), always once or twice (mean 1.45 ± SE 0.11 occurrences) during the entire association (female-unflanged: 0.022^−h^ [interactions per association hour] [Suaq]; 0.025^−h^ [Tuanan]; female-flanged: 0.016^−h^ [Suaq]; 0.011^−h^ [Tuanan]). Male aggression towards females outside of the sexual context was observed in 7.4 ± SE 0.9% of all dyadic male-female associations. Physical aggression by males directed at females was rare (Suaq: in 1 out of 393 associations; Tuanan: 9 of 521 associations) and consisted exclusively of coercive hand holding (van Schaik et al. 2006). Flanged males were significantly more likely to direct non-physical aggression in the form of displays, displacements, or short chases towards females both at Tuanan (14.0 ± SE 2.2% [0.091^−h^]) and at Suaq (9.0 ± SE 2.6% [0.025^−h^]) than unflanged males (Tuanan: 6.6 ± SE 1.5% [0.030^−h^]; Suaq: 6.6 ± SE 1.5% [0.013^−h^]) (STable 6). At Suaq and Tuanan both forced and unforced copulations were more frequent during associations involving unflanged males (Suaq: 23.5 ± SE 2.6% [0.075^−h^] [forced: 17.3 ± SE 2.3% (0.052^−h^)]; Tuanan: 15.0 ± SE 2.1% [0.041^−h^] [forced: 7.3 ± SE 1.5% (0.021^−h^)]) than flanged males (Suaq: 5.7 ± SE 2.1% [0.014^−h^] [forced: 4.1 ± SE 1.8% (0.011^−h^)]; Tuanan: 6.2 ± SE 1.5% [0.018^−h^] [forced: 1.2 ± SE 0.7% (0.005^−h^)]) (for more details: Kunz 2020). Moreover, especially unflanged males at Tuanan frequently investigated the genitals of females during associations (female-unflanged associations: 27.4 ± SE 2.4% [Tuanan], 7.7 ± SE 1.9% [Suaq]; female-flanged associations: 1.3 ± SE 0.7% [Tuanan], 4.2 ± SE 2.1% [Suaq]). These genital investigations occurred independent of the female’s offspring age (for details see suppl. mat. STable 7; SFig. 3). In summary, both affiliative and agonistic social interactions were rare during male-female associations (predictions 1.4 + 1.5, Table 1), indicating that costs of association to females likely result from increased feeding competition rather than the accompanying social interactions. However, sexual interactions were on average the most frequent social interactions during male-female associations indicating male mating effort, thus supporting prediction 1.6 and 3.2 (Table 1).

### Activity budget changes

#### Active time

**Fig. 4:**
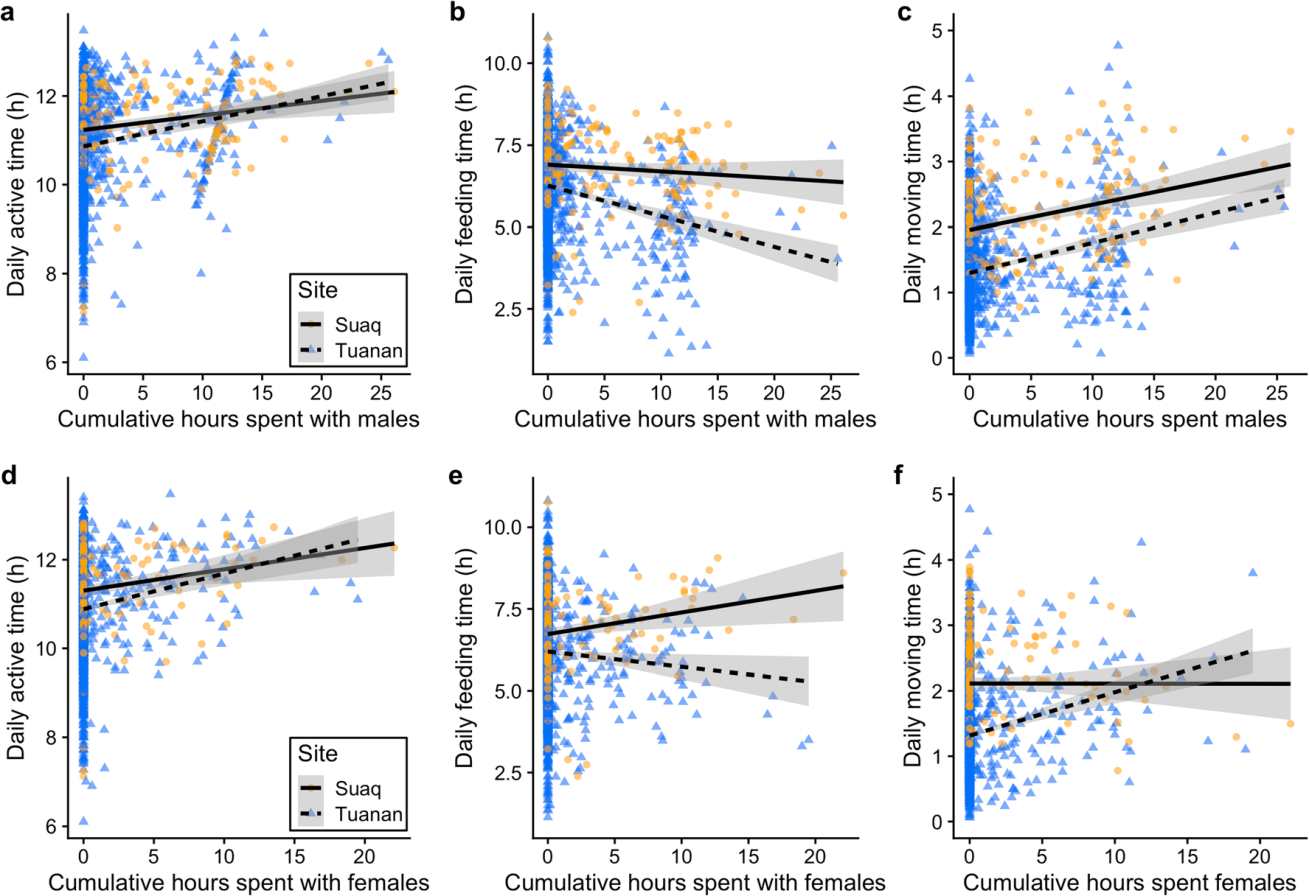
Daily female activity budget changes (*from left to right*: active time (**a**, **d**), feeding (**b**, **e**), and moving (**c**, **f**) hours) depending on cumulative hours spent with males (**a**–**c**) and females (**d**–**f**) and by study site (*round*, *orange*: Suaq; *triangles*, *blue*: Tuanan). Each data point represents one full-day focal follow (*N* = 2086), the regression lines are the correlations between hours spent with males/females and activity hours and do not show model predictions. The shaded areas display 95% confidence intervals

**Fig. 5:**
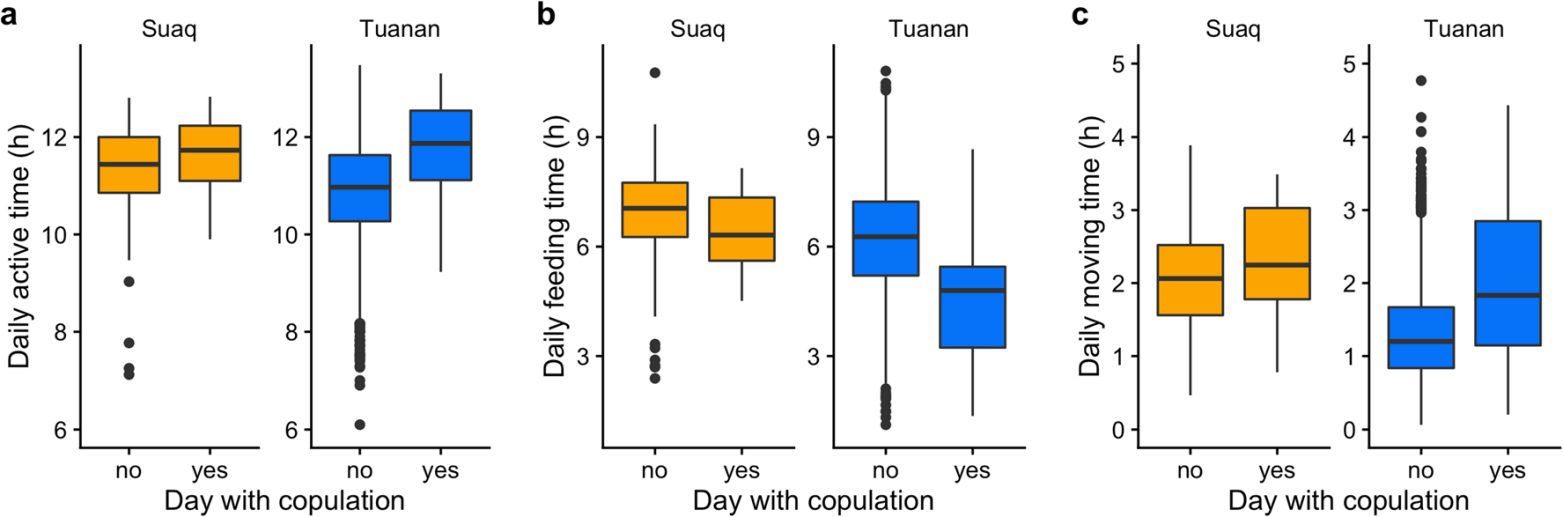
Daily female activity budget changes (*from left to right*: active time (**a**), feeding (**b**), and moving (**c**) hours) depending on the occurrence of copulations and by study site (*orange* Suaq, *blue* Tuanan). The boxplots are based on median values of full-day focal follows (*N* = 2086) and do not show model predictions (the hinges extend to the first and third quantiles and the whiskers to the largest value, and lowest, respectively, at most 1.5*inter-quartile range. Data points beyond the end of whiskers are plotted individually [Wickham 2016])

Female active time on days without any association partners except for her dependent offspring was on average 10.8 ± SD 1.0 h (min 6.1 and max 13.1), whereas on days with female associates it increased to 11.4 ± SD 1.0 h (Suaq: 11.6 ± SD 0.8; Tuanan: 11.3 ± SD 1.0) and on days with males in association (independent of association duration) to 11.4 ± SD 1.0 h (Suaq: 11.5 ± SD 0.8; Tuanan: 11.3 ± SD 1.0) (suppl. mat. STable 9). Accordingly, in both study populations, female active time increased significantly with increased time in association with females (*β* = 0.084 ± 0.025, t = 3.428, *P* = 0.001) and with males (*β* = 0.068 ± 0.034, *t* = 2.004, *P* = 0.045) (Fig. 4a+d). Moreover, at Tuanan a female’s active time increased significantly on days with copulations whereas it did not at Suaq (suppl. mat. STable 10), as the significant interaction between study site and days with copulations indicates (*β* = 0.387 ± 0.158, *t* = 2.444, *P* = 0.02) (Fig. 5a). Active time further increased with increased number of consecutive days with males (*β* = 0.056 ± 0.024, *t* = 2.385, *P* = 0.02), the total time spent in social interactions with any social partner (*β* = 0.556 ± 0.155, *t* = 3.587, *P* < 0.001), and the local fruit availability (*β* = 0.095 ± 0.039, *t* = 2.427, *P* = 0.02). Interaction terms between site and any other social factors, except copulation occurrence, did not significantly improve the model fit. In sum, daily active time increased in both populations for females in associations, and at Tuanan on days with copulations, and accordingly, the model fit significantly improved when including social factors (*χ*^2^_8,16_ = 56.27, *P* < 0.001, ΔAIC = 40.27; *N* = 2086 of 20 females and 279 FP; for the full model suppl. mat. STable 10).

#### Foraging behavior

Overall daily feeding time (F) decreased with both males and females in association, whereas moving (M) and resting time increased (Fig. 4; for detailed analyses see suppl. mat. STable 9, 11, 12). At both study sites, the F:M ratio (time spent feeding per unit moving time) of females decreased with increased association time with males (*β* = − 0.250 ± 0.053, *t* = − 4.698, *P* < 0.001), whereas it decreased significantly more at Tuanan with increased time with females in association compared to Suaq (*β* = − 0.333 ± 0.070, *t* = − 4.763, *P* < 0.001). Only consecutive days with females, but not with males, led to a further decrease in a female’s daily F:M ratio (Table 5). Furthermore, on days with copulations, the F:M ratio decreased significantly more at Tuanan than at Suaq (*β* = − 0.630 ± 0.246, *t* = − 2.557, *P* = 0.01). The full model for the F:M ratio including social factors was significantly better than the control model including ecological and physiological factors only (*χ*^2^_8,17_ = 80.47, *P* < 0.001, ΔAIC = 62.47; *N* = 2086 of 20 females and 279 FP) (Table 5). In sum, female foraging behavior was negatively affected by associations with both females and males, with the effects being more pronounced for the Tuanan population, supporting predictions 2.1 and 2.1.1 (Table 1) that costs of association arise from scramble competition of grouping. Less support was found for predictions 2.2 and 2.2.1 proposing additional costs caused by (agonistic) social interactions (Table 1).

**Table 5:** LMM output of the full model for daily feeding hours (F:M ratio) (*N* = 2086 full-day follows of 20 parous females and 279 follow periods; *χ*^2^_8,17_ = 80.47, *P* < 0.001, ΔAIC = 62.47). All fixed and control effects with *P* < 0.05 are indicated in bold. *z*, fixed effect variable was *z*-transformed prior to analysis; *O*, offset term; *C*, control factor; *F*, fixed effect

		Estimate	SE	*t*	*P*
Intercept		4.908	0.240	-	-
Moving time (h)	*Offset*				
Site (Suaq vs. Tuanan)	C	− 0.142	0.262	-	-
z Cumulative female association hours	F	0.181	0.063	-	-
**z Cumulative male association hours**	**F**	− 0.250	0.053	**− 4.698**	**< 0.001**
**Number of consecutive days with females**	**F**	− 0.167	0.070	**− 2.376**	**0.018**
Number of consecutive days with males	F	0.037	0.037	0.993	0.321
Number of copulations	F	0.193	0.206	-	-
Male-female cumulative aggression index	F	− 0.052	0.140	− 0.371	0.710
Female-female agonistic interactions (no vs. yes)	F	− 0.549	0.312	− 1.762	0.078
**z Fruit Availability Index**	**C**	− 0.150	0.063	**− 2.387**	**0.018**
z Age of dependent offspring (years)	C	− 0.047	0.069	− 0.678	0.498
**Social interaction time (h)**	**C**	− 1.045	0.241	**− 4.330**	**< 0.001**
**Site (Suaq vs. Tuanan): z cumulative female association hours**	**F**	− 0.333	0.070	**− 4.763**	**< 0.001**
**Site (Suaq vs. Tuanan): number of copulations**	F	− 0.630	0.246	**− 2.557**	**0.011**

### FCM levels

**Fig. 6:**
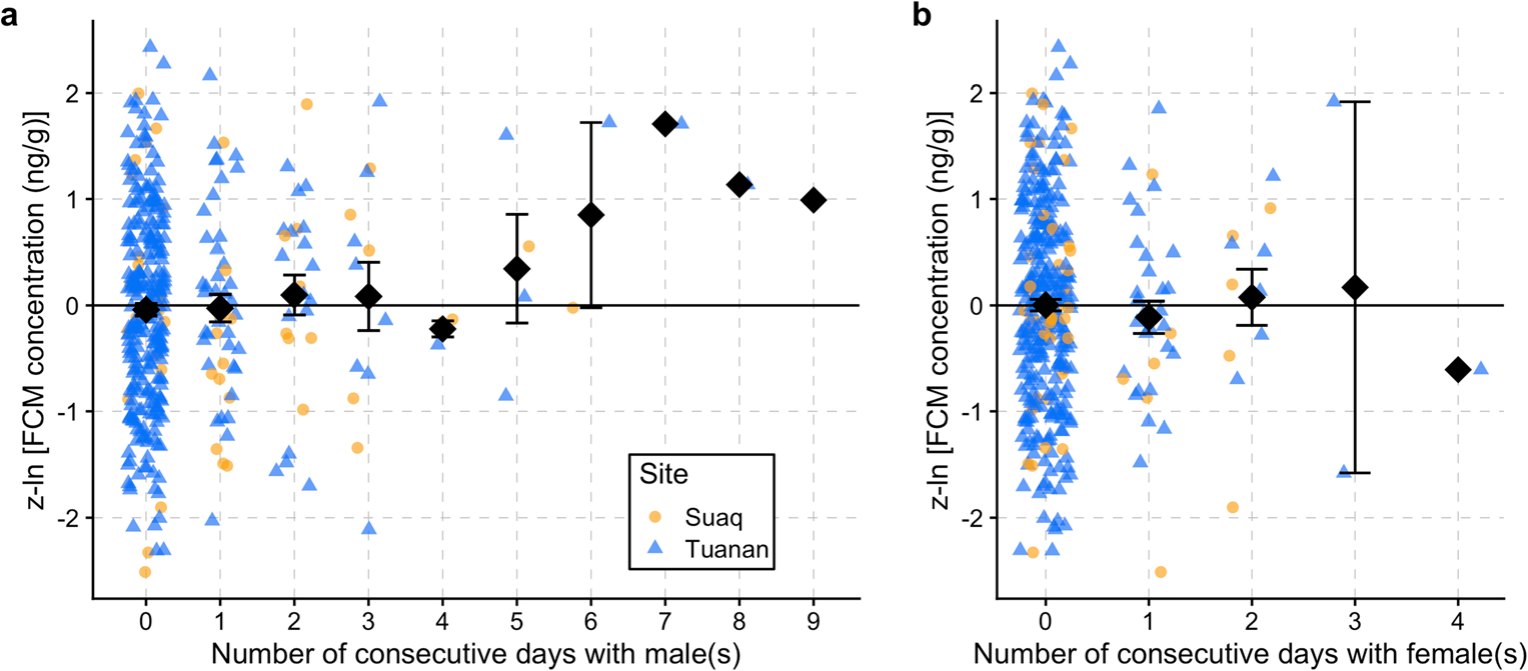
Standardized FCM levels (*z*-ln [FCM concentration (ng/g)]) (*y*-axis) of females in response to consecutive association days with adult males (**a**) and to consecutive association days with adult females (**b**). A jitter function was added to the plot to visualize the overlapping data points (consecutive days are only integers). The black diamond-shaped points indicate the mean FCM levels with the error bar (SE) in black. Study sites indicated by *round*, *orange*: Suaq; *triangles*, *blue*: Tuanan

Female FCM levels increased with the number of consecutive days in association with a male (*β* = 0.136 ± 0.047, *t* = 2.870, *P* = 0.004; Fig. 6; Table 6), but not with females (*β* = − 0.082 ± 0.085, *t* = − 0.960, *P* = 0.34). None of the other social factors, including daily association time with either females or males and the occurrence of aggression, further improved the model fit. Accordingly, the control model containing all physiological and ecological factors was improved significantly when adding the number of consecutive days with males (*χ*^2^_12,13_ = 7.30, *P* = 0.007, ΔAIC = 5.30). Although one particular female (Desy), who had male associations over a course of 9 days, appeared to be the main driver for this result, there still was a trend for consecutive days with males leading to elevated FCM levels when this female was excluded (*β* = 0.106 ± 0.056, *P* = 0.06, *N* = 333 of 89 follow periods; comparison to control model: *χ*^2^_12,13_ = 3.47, *P* = 0.06).

**Table 6:** LMM output for female FCM level changes [*z*-ln (FCM concentration (ng/g))] in response to various ecological, physiological and social factors (comparison to control model [containing only ecological and physiological factors]: *χ*^2^_12,16_ = 9.13, *P* = 0.06, ΔAIC = 1.13, *N* = 370 of 96 FPs; comparison to null model: *χ*^2^_3,16_ = 16.09, *P* = 0.24, ΔAIC = 9.91). All fixed and control effects with *P* < 0.05 are indicated in bold. *z*, fixed effect variable was *z*-transformed prior to analysis; *C*, control factor; *F*, fixed effect

	Type	Estimate	SE	*t*	*P*
Intercept		− 0.193	0.390		
Site (Suaq vs. Tuanan)	C	− 0.021	0.281	− 0.077	0.939
Consecutive days in association with female(s)	F	− 0.082	0.085	− 0.960	0.338
**Consecutive days in association with male(s)**	F	0.136	0.047	**2.870**	**0.004**
Male-female cumulative aggression index	F	− 0.110	0.118	− 0.932	0.352
Female-female agonistic interactions (no vs. yes)	F	0.063	0.443	0.142	0.887
Number of days followed	C	− 0.002	0.018	− 0.141	0.888
z Daily feeding proportion	C	− 0.072	0.054	− 1.325	0.186
z Age of dependent infant (years)	C	0.007	0.121	0.059	0.953
z Fruit Availability Index	C	0.057	0.076	0.739	0.462
Hour of sample collection	C	0.004	0.025	0.151	0.880
z Days to sample extraction	C	0.067	0.082	0.812	0.419
Laboratory (DPZ vs. IPB)	C	0.027	0.202	0.133	0.894
Total samples available with behavioral reference	C	0.005	0.008	0.560	0.577

Because our FCM data set only contained data for at most 4 consecutive days of female-female associations, we restricted the data set to sample days of at most 4 consecutive male-female association days in a further analysis. Then, the effect of consecutive days in association with males on female FCM levels was no longer significant (*β* = 0.083 ± 0.061, *P* = 0.18, *N* = 361 of 96 follow periods). In sum, it appears that only prolonged male-female associations over more than four consecutive days lead to increased female FCM levels.

## Discussion

### Foraging costs of association

Because female reproductive success is generally directly linked to access to resources (chimpanzees: Emery Thompson et al. 2007; apes: Emery Thompson et al. 2008; Stumpf et al. 2008; orangutans: Knott et al. 2009), the energetic costs of association with conspecifics have been held responsible for the varying degrees of gregariousness across the orangutan geographic distribution (van Schaik 1999). The females in our study likely suffer energetically from associations (with both males and adult females): In both study populations, females increased the length of their active day, but their feeding time decreased, both absolutely (suppl. mat. STable 12) and relative to moving time. This reduction is not only a trade-off directly resulting from increased time spent in social interactions, because (1) we controlled for time spent in social interactions, and (2) in the more sociable Sumatran population with higher forest productivity, feeding time was less affected by time spent in association with females. Hence, the reduced F:M ratio and the increased active time can be taken as direct evidence for elevated scramble competition, indicating that associations incur energetic costs to females, whereas we only found limited evidence for costs resulting from (agonistic) social interactions. We can conclude that females modify their activity budgets when in association with both males and females, in patterns that are congruent with increased scramble competition. However, to more directly assess the energetic costs of sociality in orangutans, our measure of F:M ratio should be complemented by more accurate measures of actual energy intake and measures of energy balance, such as analysis of urinary C-peptide concentrations (e.g., Emery Thompson and Knott 2008).

Orangutan females likely do not gain direct benefits from associations with males, whereas males need associations with females to monitor their reproductive status. First, genital investigations by males and male-initiated sexual interactions were the most frequent social interactions observed during male-female associations, whereas affiliative social interactions were extremely rare. However, benefits for females by associating with certain (flanged) males, such as protection from harassing males, cannot conclusively be ruled out (Mesnick 1997; Fox 2002). Second, most associations were both male-initiated and male-maintained, regardless of female reproductive state, i.e., the age of the female’s dependent offspring, and females likely incurred costs from those involuntary associations as discussed above. When females are ready to conceive, however, they may actively seek the association with (dominant) flanged males (Fox 1998, 2002; Spillmann et al. 2010). With our analyses, we did not capture this short window around conception. We conclude that females and males are likely at odds about association maintenance. Accordingly, orangutan females have been reported to actively avoid male associates or try to end associations as rapidly as possible (Fox 2002; Mitra Setia and van Schaik 2007; Utami Atmoko et al. 2009; van Noordwijk and van Schaik 2009; Spillmann et al. 2010; Knott et al. 2018). Further investigations to understand how and if females attempt to avoid male associates have to be conducted, including the analysis of simultaneous ranging data. In sum, our study indicates that females incur costs from male-maintained associations, but no clear immediate benefits (albeit perhaps indirect ones: Kunz 2020), especially during the period of lactational infertility (~ 6.5 years [van Noordwijk et al. 2018]). These costs of involuntary associations may be relevant, because orangutan females’ reproductive success highly depends on the availability of resources (Knott et al. 2009), particularly in a less productive habitat (Wich et al. 2011).

### Stress and association

Female FCM levels increased as they spent more days in association with males, but not with females. This social factor was the best and only predictor for FCM level changes. Thus, repeated days of increased active time and reduced F:M ratio led to a physiological stress response. Interestingly, this was not the case when in association with other females, because females can avoid lengthy associations with other females before associations become too costly. Conversely, males appear to profit from associations with females and they maintain associations over a longer time period than a female would. The behavioral data available support this conclusion: Female-female associations never lasted more than 4 consecutive days at Tuanan, where the F:M ratio decreased significantly more when in association with other females than at Suaq, while male-female associations could last up to 8 days. The elevated FCM levels of captive orangutan females when artificially confined to permanent association with males (Amrein et al. 2014) further support our hypothesis that increased sociality over an extended time period leads to a physiological stress response, especially in Bornean orangutans. The findings in captivity suggest that Bornean females show stress reactions to extended sociality even in the absence of reduced net energy intake, suggesting that in captivity increased FCM levels in females associated with males more likely reflect social rather than energetic stress. Although our endocrine data set is very limited, especially for the extended consecutive association days with males, we propose that only extended association periods with males lead to increased FCM levels as seen in captivity. However, whether these FCM elevations observed in our wild females are a response to the association itself or, alternatively, reflect energetic constraints due to the association-related decrease in feeding time and increase in active time is unclear. Future studies should generally aim at obtaining a more conclusive endocrine data set including larger sample sizes linked to consecutive association days.

Following the same line of argument, one would expect to find more pronounced FCM level changes in the less sociable Bornean orangutans in response to involuntary associations compared to Sumatran orangutans. Although we could not find any evidence for differences in FCM level changes between Suaq and Tuanan, our data set was very small for the Suaq population (*N* = 52 samples, a maximum of 6 [known] consecutive days in male-female association). Thus, the comparison should be repeated with a more extensive data set in the future. A difference in the physiological response to social stressors, including energy balances, may be expected in the light of the socioecological theory, because the degree of sociability between the two populations differs (this study; van Schaik 1999). Since our activity and feeding data indicate that both associations (with females) and social interactions are costlier to Tuanan females than to Suaq females, where fruit availability is generally higher (Wich et al. 2011), a stronger physiological stress response would be expected at Tuanan. Future studies are, however, needed to test this hypothesis and thus to evaluate whether females of the more sociable Sumatran orangutan may be more “stress-resistant” which could explain why there is less need for either behavioral or physiological mechanisms to avoid associations.

We found no evidence for differences in female FCM levels on days with any agonistic interaction with either males or females in the two populations. Even though days with copulations were characterized by increased active time and reduced F:M ratio at Tuanan, we found no evidence that male aggression, in particular sexual coercion (SFig. 7), imposed any additional costs, either as reduced feeding time or in elevated FCM levels. If these forced copulations are cost insensitive, they would not qualify as sexual coercion by the definition of Smuts and Smuts (1993) (“use by a male of force, or threat of force, that functions to increase the chances that a female will mate with him at a time when she is likely to be fertile, and to decrease the chances that she will mate with other males, at some cost to the female”), while prolonged, male-maintained associations would. However, the absence of a stress response does not exclude other costs of forced copulations, such as the limitation to the expression of female mating preferences. Indeed, the consistent attempts by females to escape from involuntary mating initiations (Fox 2002; Knott et al. 2010) suggest that females perceive resisted copulations as undesired rather than as a way to assess mate quality. For now, therefore, interpreting forced copulations as sexual coercion remains the most plausible explanation.

Since fecal cortisol metabolite levels represent an integrative measure of pooled endocrine activity over several hours or days (Hodges and Heistermann 2011), it is likely unsuited to detect short-term stress responses to a specific behavioral event. Forced copulations lasted on average 8.8 ±SD 7.2 min (Kunz 2020), and any stress response associated with this behavior is likely to be too short to be detected by our FCM measure. Urinary cortisol levels may thus be a more appropriate measure to assess whether particular social interactions induce more immediate elevations in cortisol production (e.g., Silk et al. 2013) as has been shown for chimpanzees (Muller et al. 2007; Emery Thompson et al. 2010). Further detailed studies, with a larger sample size and more immediate measures of cortisol levels from urine, are needed to examine whether female orangutans do indeed not show stress responses to forced copulations.

### The male perspective

Both unflanged and flanged males are responsible for maintaining associations, independent of the females’ dependent offspring age (as a proxy for reproductive state), which supports the hypothesis that the males’ interest to associate exceeds that of the females (Table 1). Besides mating opportunities, these associations may be an attempt to monitor a female’s reproductive state and sexual activities. In the absence of any apparent signal of fertility (Nunn 1999), it remains uncertain how males assess female reproductive state, if at all. The genital investigations reported here may provide some olfactory information to males (cf. chimpanzee: Matsumoto-Oda et al. 2003; review: Drea 2015), but data are insufficient to know how and if these relate to sexual interactions (suppl. mat. STable 8). It is likely that males also incur energetic costs from associations and interactions with females (cf. East African chimpanzees [*P. troglodytes schweinfurthii*]; Emery Thompson and Georgiev 2014; Georgiev et al. 2014), and our unpublished data suggest this, too, for orangutan males. Thus, males may have a set of decision rules when and for how long to associate with certain females. Accordingly, the time in association with males increases with the increasing age of the dependent offspring of females (this study; van Schaik 1999), suggesting some type of reproductive benefits for males. More detailed analyses on the social context of associations will provide further insight into how males benefit from sociality with females.

It remains to be investigated if prolonged male-maintained associations should be labelled as a separate indirect form of sexual coercion or may even function as coercive mate guarding, i.e., “to constrain female promiscuity” (Muller et al. 2009). First, direct non-sexual aggression towards females by males is rare in orangutans (STable 6; SFig. 2) providing little evidence for any herding, punishment or sequestration (apart from the ten cases of coercive hand holding). However, anecdotal data suggest subtle sequestration attempts, in that males may try to influence females’ travel direction away from other males during associations (MAvN et al., unpubl. data). Second, copulations regularly occur in the presence of other, even more dominant, males (Fox 2002). (Coercive) mate guarding by males therefore appears to be an inefficient strategy, especially for subordinate, unflanged males. Third, although we found evidence for direct costs for females resulting from male-maintained associations, which indicates male coercion, we cannot rule out that females ultimately benefit indirectly from paternity confusion through those male-driven association patterns. Future studies are needed to evaluate the social contexts of associations.

## Conclusion

Here, we report evidence for sexual conflict over associations in orangutans. We conclude that females incur costs from male-maintained associations, especially if those associations last multiple days. The costs include reduced feeding time and increased moving and resting time, which adds up to longer activity per day and thus shorter night rest. Furthermore, prolonged associations with males were associated with elevated FCM levels, whereas this was not the case for female-female associations which were usually much shorter. We suggest that the absence of morphological fertility advertisement in female orangutans may be explained by these costs of association, thus supporting the first prediction of the “cost-of-sexual-attraction” hypothesis (Wrangham 2002) for orangutans. The length of sexual attractivity negatively correlates with the cost of association for females in the genus *Pan* (Wrangham 2002). Orangutans fit into this fission-fusion continuum at the solitary end: They do not exhibit any morphological signal of fertility, arguably because this would attract too many competing males at once leading to a prolonged period of unacceptably high energetic costs for the females, in addition to the mere physiological costs associated with the swelling itself (for a review: Nunn 1999). On the contrary, female orangutans advertise non-availability with small labial swellings during pregnancy (Schultz 1938; Galdikas 1981), likely to reduce the costs of association as males refrain from maintaining associations and copulating with pregnant females exhibiting the labial swelling (only 2 out of 34 pregnancy matings were observed when females exhibited a pregnancy swelling, JAK et al. unpubl. data).

Yet, females of both *Pan* spp. and *Pongo* spp. exhibit unpredictable ovulation, albeit to varying extent (Nadler 1981; Deschner et al. 2004; Douglas et al. 2016), which has been linked to paternity confusion serving infanticide avoidance strategies (Hrdy 1979; Hrdy and Whitten 1987; van Schaik et al. 2004). The concealed ovulation in orangutans (Nadler 1981) may therefore also serve to reduce the risk of infanticide as it does in most other primates (Hrdy 1979; van Schaik et al. 2004). Female orangutans seem to vary their mate preferences with their reproductive status accordingly (Knott et al. 2010). However, evidence for infanticidal attacks by males remains indirect (Beaudrot et al. 2009; Knott et al. 2019; Scott et al. 2019) and infant mortality is generally extremely low (van Noordwijk et al. 2018), suggesting that male infanticide in orangutans is extremely rare compared to chimpanzees and that females employ efficient counterstrategies.

In a dispersed mating system with high costs of association, and where males generally drive association patterns as found here for orangutans, the lack of morphological fertility advertisement can be explained by the selection on the total concealment of ovulation. Given a risk of infanticide (Knott et al. 2010, 2019), females must achieve an optimum distribution of paternity assessments (van Schaik and Janson 2000; van Schaik et al. 2004) by removing as much information on female fertility status as possible. Accordingly, the absence of morphological fertility advertisement combined with the concealed ovulation in orangutans appears to be the result of a trade-off between the costs of association and the necessity for paternity confusion (van Schaik et al. 2004; Knott et al. 2010, 2019). Future work will have to elaborate on the details of this hypothesis.

## Supplementary information

ESM 1 (DOCX 1.09 MB)

## Data Availability

The main data sets generated and analyzed during the current study are available in the Harvard Dataverse repository, 10.7910/DVN/WXDVF6.
