# Supplementary material for: The cost of associating with males for Bornean and Sumatran female orangutans: a hidden form of sexual conflict?
Source: Behav Ecol Sociobiol. 2020 Dec 30;75(1):6. doi: 10.1007/s00265-020-02948-4 (PMC7773621; doi:10.1007/s00265-020-02948-4)
Supplement: Supplementary file 1 — (DOCX 1.09 MB) [file 265_2020_2948_MOESM1_ESM.pdf]

Behavioral Ecology and Sociobiology

The cost of associating with males for Bornean and Sumatran female orangutans: a hidden form of sexual conflict?

Julia A. Kunz[1]*, Guilhem J. Duvot[1], Maria A. van Noordwijk[1], Erik P. Willems[1], Manuela Townsend[1], Neneng Mardianah[2], Sri Suci Utami Atmoko[2], Erin R. Vogel[3], Taufiq Purna Nugraha[4,5], Michael Heistermann[6], Muhammad Agil[5], Tony Weingrill[1], Carel P. van Schaik[1]

1) Department of Anthropology, University of Zurich, Switzerland

2) Faculty of Biology and Primates Research Center, Universitas Nasional, Indonesia

3) Department of Anthropology, Rutgers The State University of New Jersey, USA

4) Research Center for Biology, Indonesian Institute of Sciences (LIPI), Cibinong, Indonesia

5) Faculty of Veterinary Medicine, Bogor Agriculture University, Indonesia

6) Endocrinology Laboratory, German Primate Center, Leibniz-Institute for Primate Research, Germany

* Corresponding author: juliaandrea.kunz@uzh.ch

Supplementary Material

Association patterns

Association frequency

Stable 1 LMM output for average daily association hours that parous females spent with a) females, b) unflanged males and c) flanged males depending on study site, FAI, the age of their dependent infant (years, as proxy for their reproductive state) and number of days available per follow period (FP) (N = 279 FP [>4 full-day follows] of 20 parous females [random intercept]). Fixed factors with P < 0.05 are indicated in bold

Response	Fixed effect	Type	Estimate	SE	t	P
a) Daily hours with parous females	Intercept		1.224	0.364		
	**Site (Suaq vs. Tuanan)**	Control	-1.058	0.360	**-2.939**	**0.008**
$\chi^2_{5,7} = 1.12$, P = 0.57,	z Fruit Availability Index	Fixed	-0.007	0.061	-0.116	0.907
ΔAIC = 2.88	z Age of dependent offspring (y)	Fixed	0.067	0.063	1.066	0.288
	Number of days per FP	Control	0.023	0.025	0.930	0.353
b) Daily hours with unflanged males	Intercept		2.461	0.620		
	**Site (Suaq vs. Tuanan)**	Control	-1.821	0.556	**-3.273**	**0.003**
$\chi^2_{5,7} = 27.26$, P < 0.0001,	**z Fruit Availability Index**	Fixed	0.265	0.123	**2.146**	**0.033**
ΔAIC = 23.26	**z Age of dependent offspring (y)**	Fixed	0.606	0.126	**4.799**	**<0.001**
	Number of days per FP	Control	0.031	0.050	0.625	0.532
c) Daily hours with flanged males	Intercept		0.598	0.404		
	Site (Suaq vs. Tuanan)	Control	0.228	0.332	0.688	0.495
$\chi^2_{5,7} = 28.04$, P < 0.0001,	z Fruit Availability Index	Fixed	0.166	0.088	1.880	0.061
ΔAIC = 24.04	**z Age of dependent offspring (y)**	Fixed	0.448	0.090	**5.001**	**<0.001**
	Number of days per FP	Control	-0.046	0.036	-1.284	0.200

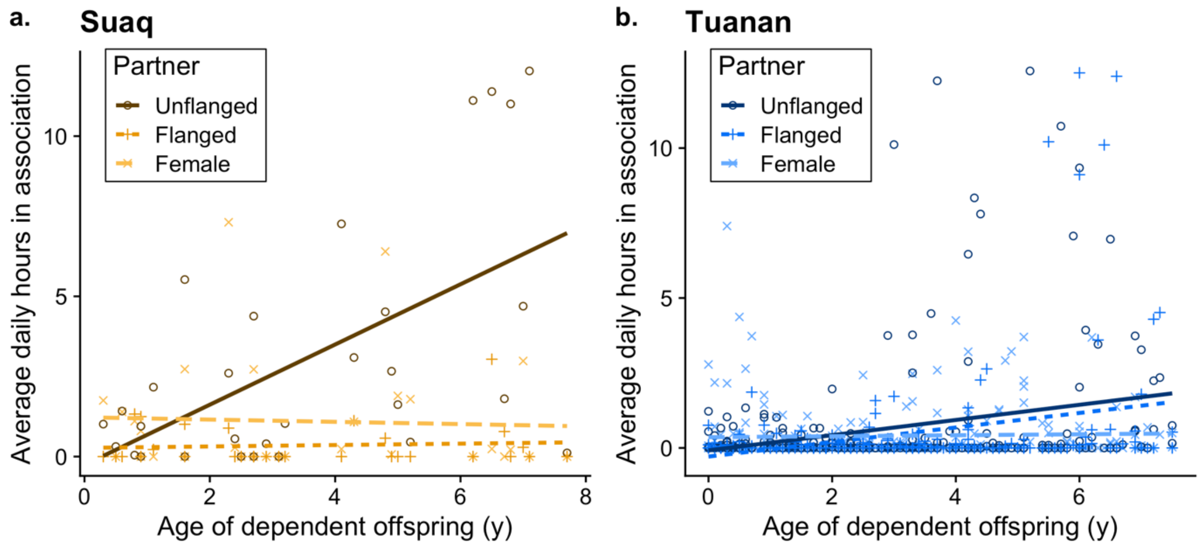

Sfigure 1 Average daily hours that parous females spent in association with other parous females, unflanged and flanged males by study site and depending on the age of their dependent offspring (years). Each data point is based on the average of a female follow period (N = 279), which consists of at least 5 full-day focal follows.

Association maintenance

Stable 2 GLMM output for the probability that a dyadic association was male-maintained (FHI < 0) by study site, male morph, age of the dependent offspring (years), copulation occurrence, association duration, and fruit availability. Fixed factors with P < 0.05 are indicated in bold

	Estimate	SE	Odd's ratio	z	P
Intercept	-0.097	0.215			
Site (Suaq vs. Tuanan)	-0.024	0.250	1.02	0.095	0.924
**Male morph (unflanged vs. flanged)**	0.653	0.201	1.92	**3.244**	**0.001**
z Age of dependent infant (y)	0.089	0.100	1.09	0.891	0.373
Occurrence of copulation (no vs. yes)	0.556	0.298	1.74	1.866	0.062
**z Association duration (h)**	1.226	0.255	3.40	**4.804**	**<0.001**
z Fruit Availability Index	-0.019	0.089	0.98	-0.215	0.829
$\chi^2_{3,9} = 72.53$, P < 0.001, ΔAIC = 60.53					
N = 665 of 30 female and 140 male identities					

Association maintenance over consecutive days

The survival analysis on the association maintenance over consecutive days was run twice. Once including the associations with unknown start (N = 625 associations, reported in the main text) and once excluding them (N = 446). The associations with unknown start were included in the analysis, because the start is less likely to be unknown for long associations and by excluding them we would have biased our analysis against such long associations (Stable 3). However, the exclusion of the associations with unknown start does not lead to a different pattern (Stable 4).

Stable 3 Output of the binomial GLMM for the probability that the start of an association is known depending on study site, association partner sex and the total duration of the association (days). Fixed factors with P < 0.05 are indicated in bold

	Estimate	SE	z	P
Intercept	1.237	0.342		
Site (Suaq vs. Tuanan)	0.482	0.298	1.618	0.106
Association partner sex (female vs. male)	0.067	0.245	0.273	0.785
**Number of days in association**	-0.386	0.108	**-3.594**	**<0.001**
$\chi^2_{3,6} = 17.26$, P = 0.0006				
N = 625 associations of 21 females and 168 different follow periods				

Stable 4 Probability of ending an association: Output of the Cox proportional hazard mixed model for the total number of (known) days in association by the type of association partner, study site, age of the dependent offspring (years), and zFAI (N = 446 associations of which 296 with known end; 20 female identities and 148 FPs). Only associations with known start are included in the analysis. Fixed factors with P < 0.05 are indicated in bold

	coef	SE	Hazard ratio	z	P
Association partner					
Sex (male vs. female)	0.274	0.091	1.315	-	-
Male morph (unflanged vs.flanged)	0.065	0.130	1.067	-	-
Site (Suaq vs. Tuanan)	0.563	0.185	1.756	-	-
Age of dependent offspring (y)	-0.126	0.082	0.882	-1.530	0.130
z Fruit Availability Index	-0.028	0.084	0.972	-0.330	0.740
**Association partner sex (male vs. female) : Site**	-0.259	0.112	0.772	**-2.300**	**0.021**
Partner male morph (unflanged vs.flanged) : Site	-0.032	0.163	0.968	-0.200	0.840
$\chi^2_{7} = 23.47$, P = 0.001					
N = 446 associations of which 296 with known end					

Social interactions during male-female associations

Social interactions among association partners are generally rare in orang-utans compared to other more gregarious primate species. Yet, male-female associations do not only occur when a female is likely fertile (Stable 1; Sfigure 1) and mating only occurred in 11.1% of all male-female associations. To evaluate what other benefits associations could have for either or both females and males, we report the social interactions that were observed during associations including any agonistic or affiliative interaction and sexual investigations.

Affiliative social interactions

Affiliative social interactions, including begging for food, food sharing, social play, and any form of grooming (independent of the directionality) was more likely in associations with unflanged than flanged males (Stable 5). Moreover, affiliative social interactions between males and females increased with the female's offspring age at Suaq, but not at Tuanan (Stable 5; Sfigure 2).

Stable 5 GLMM output for the probability of affiliative social interactions (0/1) in a dyadic association by study site, partner male morph, and the age of the dependent offspring (years) as a proxy of female reproductive state. The association duration (h), taken as all the active time a dyad was <50m, was added as an offset term to correct for the varying time in association. Fixed factors with P < 0.05 are indicated in bold

Fixed effects	Estimate	SE	Odd's ratio	z	P
Intercept	0.362	0.406			
Association duration	*Offset term*				
**Male morph (unflanged vs. flanged)**	-1.061	0.472	0.35	**-2.248**	**0.025**
Site (Suaq vs. Tuanan)	-0.541	0.532	0.58	-	-
z Age of dependent offspring (y)	0.907	0.378	2.48	-	-
**Site : z Age of dependent offspring (y)**	-1.457	0.454	0.23	**-3.211**	**0.001**
$\chi^2_{3,7} = 20.63$, P = 0.0004, ΔAIC = 12.63					
N = 922 associations of 32 parous female IDs and 190 male IDs					

Male aggression towards females

Besides the most obvious form of sexual coercion, forced copulation, male aggression towards females in orangutans is rare and especially physical aggression hardly occurs outside of the sexual context. The occurrence of male aggression, including displays, displacement events and chases, was more frequently observed by flanged than unflanged males (Stable 6; Sfigure 2). Moreover, male aggression decreased with the increasing age of the dependent offspring (Stable 6).

Stable 6 GLMM output for the probability of male aggression directed at females (0/1) during dyadic associations by study site, male morph and the age of the dependent offspring (years) as a proxy of female reproductive state. The association duration (h), taken as all the active time a dyad was <50m, was added as an offset term to correct for the varying time in association. Fixed factors with P < 0.05 are indicated in bold

Fixed effects	Estimate	SE	Odd's ratio	z	P
Intercept	-0.252	0.332			
Association duration (h)	*Offset term*				
Site (Suaq vs. Tuanan)	0.443	0.376	1.56	1.179	0.238
**Male morph (unflanged vs. flanged)**	1.194	0.346	3.30	**3.450**	**0.001**
**z Age of dependent offspring (y)**	-0.292	0.142	0.75	**-2.053**	**0.040**
$\chi^2_{3,6} = 15.52$, P = 0.001, ΔAIC = 9.52					
N = 922 associations of 32 female IDs and 190 male IDs					

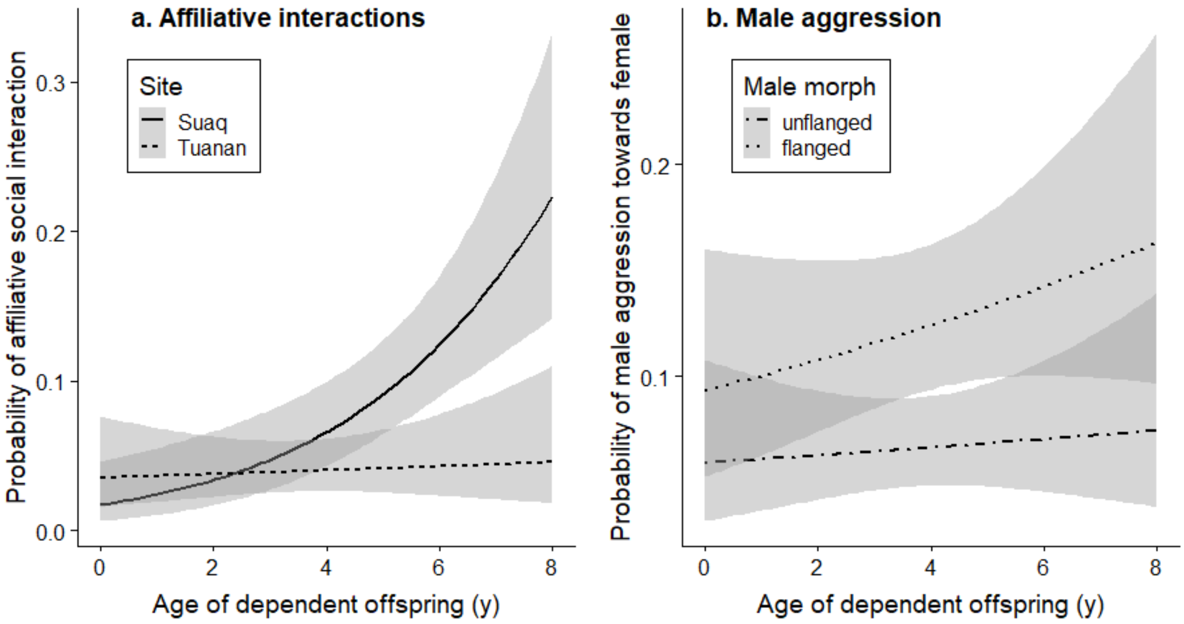

Sfigure 2 Probability of affiliative social interactions (a) and male aggression (b) in a male-female association dyad by the age of the dependent offspring of a female (years) and a) study site and b) male partner morph. The lines indicate the direct relationship between affiliation/aggression probability and the age of the dependent offspring and not the model predictions (Stab. 5 & 6). The data points are based on separate male-female association units (N = 922), which may have lasted several days.

Sexual investigations

Males were reported to investigate the genitals of females with hands and mouth and occasionally even drink the females' urine. So far, it is unknown what information the males gain from these sexual investigations. Two scenarios are possible: Males perceive either an olfactory cue indicative of female reproductive state or of previous sexual interactions, i.e. if a competitor had mated previously. Here, we evaluate in which associations these sexual investigations occur. Especially unflanged males at Tuanan investigate females frequently (almost in a third of all associations). Accordingly, the interaction between site and male morph was significant (Stable 7). At Suaq, investigation rates of unflanged and flanged males were in a similar range as that of flanged males at Tuanan (Sfigure 3). Investigations are more likely to occur in longer associations. Moreover, males already investigate the genitals of females with very young dependent offspring and the investigation probability does not change with the infants age. The question then arises how sexual investigations relate to copulations. Copulations did not always occur in associations when sexual investigations took place. Given a sexual investigation was observed, the probability that also a copulation occurred during the same association increased with the age of the dependent offspring (years) and with increasing association duration (Stable 8). The increasing copulations-sexual investigation co-occurrence with the increasing age of the dependent offspring, may be taken as an indication that males perceive some olfactory cue indicative of the female reproductive status. However, future studies including the measurements of female reproductive hormones are needed to investigate what information males gain from the genital investigations.

Stable 7 Binomial GLMM output for the occurrence of sexual investigations during dyadic male-female associations by the study site, male morph, the age of the dependent offspring (years), fruit availability and the number of other males in association. Fixed factors with P < 0.05 are indicated in bold

Fixed effects	Estimate	SE	Odd's ratio	z	P
Intercept	-2.996	0.339			
Site (Suaq vs. Tuanan)	1.941	0.350	6.96	-	-
Male morph (unflanged vs. flanged)	-0.590	0.590	0.55	-	-
z Age of dependent offspring (y)	-0.121	0.168	0.89	-0.723	0.469
z Fruit Availability Index	-0.164	0.119	0.85	-1.382	0.167
**z Association duration (h)**	0.533	0.115	1.70	**4.647**	**<0.001**
Number of other males in association	0.220	0.126	1.25	1.742	0.082
**Site * Male morph**	-2.784	0.797	0.06	**-3.494**	**<0.001**
$\chi^2_{3,10}$ = 135.26, P < 0.0001, ΔAIC = 121.26					
N = 960 associations of 31 female IDs and 174 male IDs					

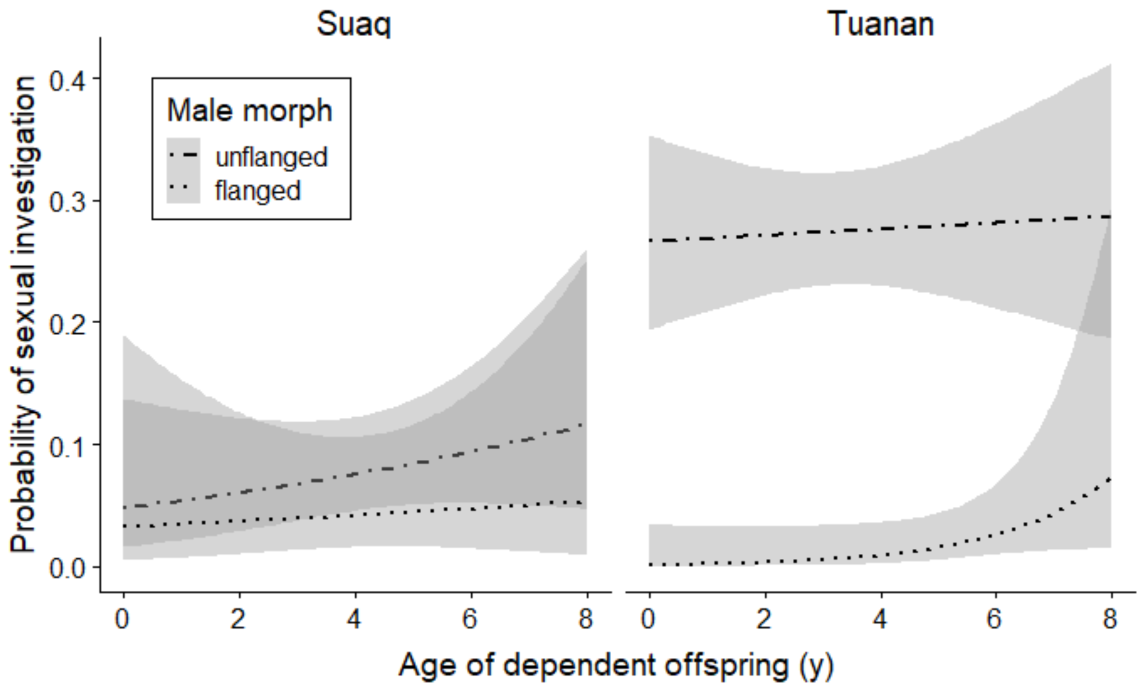

Sfigure 3 Probability of sexual investigations by males during dyadic associations with females by the female's dependent offspring age (years), study site (left: Suaq; right: Tuanan) and the male morph (dot-dashed line: unflanged; dotted line: flanged). The lines are based on the logistic correlation between the age of the dependent offspring and the occurrence of sexual investigations and do not depict model predictions.

Stable 8 Binomial GLM output for the probability of copulation occurrence given a sexual investigation was observed during a dyadic association by study site, male morph, age of the dependent offspring (years), zFAI and association duration. Fixed factors with P < 0.05 are indicated in bold

**Fixed effects**	**Estimate**	**SE**	**Odd's ratio**	**z**	**P**
Intercept	-3.128	1.095			
Site (Suaq vs. Tuanan)	0.085	0.957	1.09	0.089	0.929
Male morph (unflanged vs. flanged)	1.133	1.099	3.10	1.031	0.302
**z Age of dependent offspring (y)**	2.346	0.662	10.45	**3.543**	**<0.001**
z Fruit Availability Index	0.213	0.304	1.24	0.700	0.484
**z Association duration (h)**	1.061	0.309	2.89	**3.435**	**<0.001**

χ^2_5=70.74, P<0.0001, ΔAIC=60.74

N= 122 associations with sexual investigations

Activity budget changes

Stable 9 Overview of absolute daily female activity budget (mean daily active time, daily feeding, resting and moving hours) depending on the presence of additional adult female or male association partners (no association, adult female or male in association) by study site. Values are mean daily hours spent in specific activity and the standard deviation is indicated. Below the study site the corresponding sample size (number of female full day focal follow days) of a given category is indicated (N=…)

	**No association** *with either adult female or male*		**Adult female** *in association (yes/no)*		**Adult male** *in association (yes/no)*		**Adult male** *less than half of active time in association*		**Adult male** *more than half of active time in association*	
**Site** / **Activity**	Suaq N = 85	Tuanan N =1372	Suaq N = 50	Tuanan N =187	Suaq N =114	Tuanan N =354	Suaq N =49	Tuanan N =227	Suaq N =65	Tuanan N =127
Daily active time (h)	11.1 ± 1.0	10.8 ± 1.0	11.6 ± 0.8	11.3 ± 1.1	11.5 ± 0.8	11.3 ± 1.0	11.4 ± 0.9	11.4 ± 1.0	11.6 ± 0.8	11.3 ± 1.0
Daily feeding time (h)	6.8 ± 1.4	6.3 ± 1.4	6.9 ± 1.4	5.9 ± 1.5	6.8 ± 1.3	5.9 ± 1.6	6.9 ± 1.5	6.3 ± 1.5	6.7 ± 1.1	5.1 ± 1.5
Daily moving time (h)	1.9 ± 0.6	1.2 ± 0.6	2.2 ± 0.6	1.7 ± 0.8	2.3 ± 0.7	1.6 ± 0.8	2.1 ± 0.7	1.5 ± 0.7	2.4 ± 0.7	1.8 ± 1.0
Daily resting time (h)	1.8 ± 1.1	3.0 ± 1.3	1.8 ± 1.1	3.3 ± 1.3	1.9 ± 1.1	3.5 ± 1.6	1.9 ± 1.3	3.1 ± 1.3	1.9 ± 1.0	4.1 ± 1.9

Active time

Stable 10 Full model for daily active time (hours) by social, ecological and physiological factors. Social factors improved control model significantly ($\chi^2_{8,16}$ = 56.27, P < 0.001, ΔAIC = 40.27), whereas there was no improvement when adding an interaction term with site and time in association with males or females ($\chi^2_{16,18}$ = 0.49, P = 0.78, ΔAIC = 3.51). Fixed factors with P < 0.05 are indicated in bold. (*Note:* z = fixed effect variable was z-transformed prior to analysis; C = control factor, F = fixed effect)

		**Estimate**	**SE**	**t**	**P**
Intercept		11.094	0.154	-	-
Site (Suaq vs. Tuanan)	C	-0.168	0.170	-	-
**z Cumulative female association hours**	F	0.084	0.024	**3.428**	**0.001**
**z Cumulative male association hours**	F	0.068	0.034	**2.004**	**0.045**
Number of copulations	F	-0.025	0.133	-	-
Male-female cumulative aggression index	F	0.027	0.090	0.295	0.768
Female-female agonistic interactions (no vs. yes)	F	-0.323	0.201	-1.607	0.108
**z Fruit Availability Index**	C	0.095	0.039	**2.427**	**0.016**
z Age of dependent offspring (y)	C	0.070	0.043	1.646	0.101
**Social interaction time (h)**	C	0.556	0.155	**3.587**	**<0.001**
Consecutive days with female count	F	0.052	0.045	1.146	0.252
**Consecutive days with male count**	F	0.056	0.024	**2.385**	**0.017**
**Site : Number of copulations**	F	0.387	0.158	**2.444**	**0.015**

$\chi^2_{4,16}$ = 108.02, P < 0.001, ΔAIC = 84.02
N = 2086; 20 IDs and 279 FPs

Because the active time increased with several social factors (Stable 10), we assessed if and how the different activities changed relative to this increased active time (Stable 11) and how the activity budget changed absolutely (Stable 12).

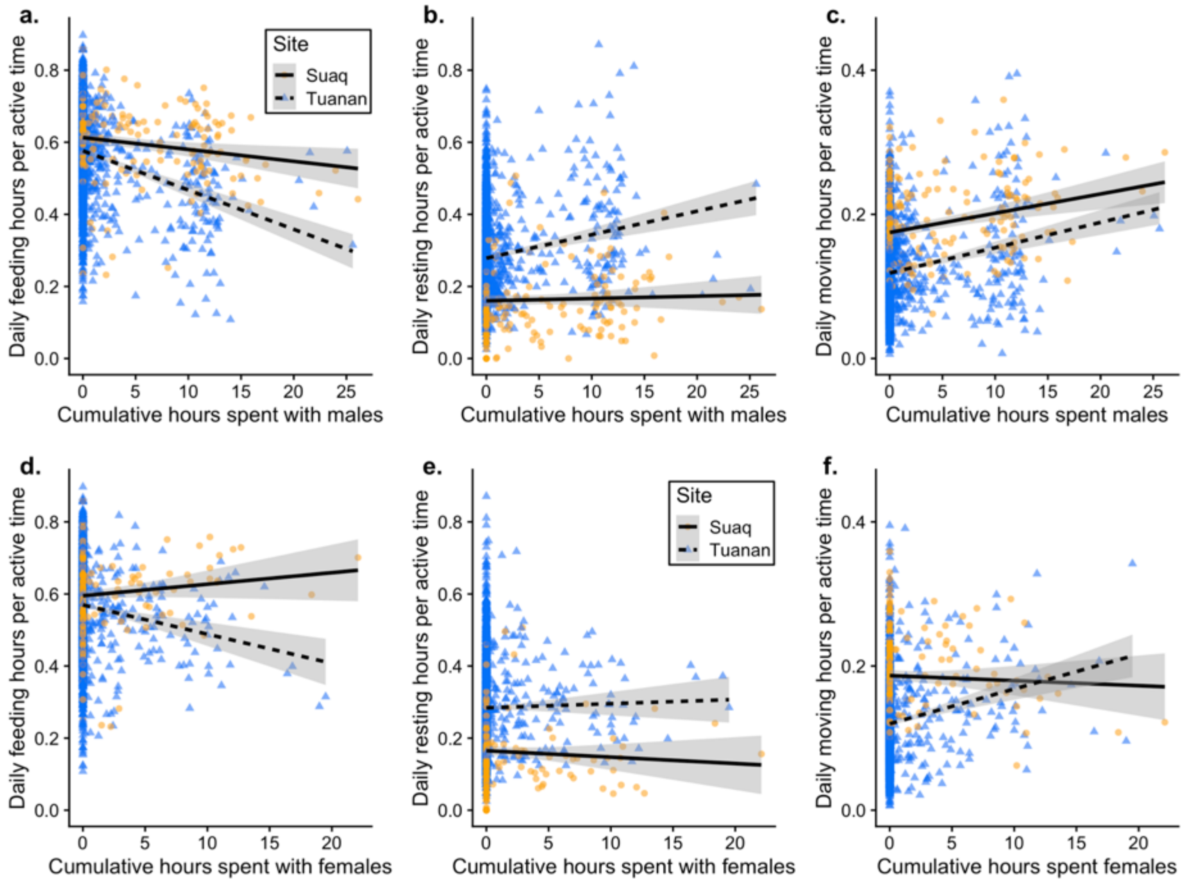

Sfigure 4 ***Daily activity budget changes*** (feeding (a, d), resting (b, e) and moving (c, f) hours [per total active hours]) depending on the cumulative hours spent in association with males (*top row:* a, b, c) and with females (*bottom row:* d, e, f) by study site (circle: Suaq; triangle: Tuanan). Data points are based on full-day focal follows of parous females from follow periods of at least 5 days. *Note:* The lines indicate the correlation between the activity and the time spent with males/females, but not the model predictions

Stable 11 LMM outputs of the full model for daily feeding, moving and resting hours of parous females (N = 2086 full-day follows of 20 parous females and 279 follow periods). Daily active time was included as an offset term in all the models. The comparison of each full model (reported model) to the control model is reported in the first column below the response variable. All fixed and control effects with P < 0.05 are indicated in bold. (*Note:* z = fixed effect variable was z-transformed prior to analysis; O = offset term; C = control factor, F = fixed effect)

Response	Fixed effects		Estimate	SE	t	P
**Feeding hours**	Intercept	I	-4.413	0.264	-	-
	Active time (h)	*Offset*				
	Site (Suaq vs. Tuanan)	C	-0.507	0.300	-	-
$\chi^2_{8,18}$ = 132.2, P < 0.001, ΔAIC =112.19	z Cumulative female association hours	F	0.097	0.054	-	-
	z Cumulative male association hours	F	-0.135	0.069	-	-
	**Consecutive days with female count**	F	-0.148	0.061	**-2.438**	**0.015**
	Consecutive days with male count	F	0.012	0.032	0.389	0.697
	Number of copulations	F	0.093	0.184	-	-
	Male-female cumulative aggression index	F	-0.157	0.122	-1.293	0.196
	Female-female agonistic interactions (no vs. yes)	F	-0.095	0.270	-0.353	0.724
	z Fruit Availability Index	C	-0.096	0.052	-1.843	0.066
	z Age of dependent offspring (y)	C	0.016	0.058	0.285	0.776
	**Social interaction time (h)**	C	-1.522	0.209	**-7.286**	**<0.001**
	**Site : z Cumulative female association hours**	F	-0.284	0.061	**-4.661**	**<0.001**
	**Site : z Cumulative male association hours**	F	-0.205	0.082	**-2.489**	**0.013**
	**Site : Number of copulations**	F	-0.611	0.225	**-2.721**	**0.007**
**Moving hours**	Intercept	I	-9.209	0.158	-	-
	Active time (h)	*Offset*				
	**Site (Suaq vs. Tuanan)**	C	-0.399	0.178	**-2.242**	**0.038**
$\chi^2_{4,8}$ = 23.17, P < 0.001, ΔAIC = 15.2	**Social interaction time (h)**	C	-0.648	0.156	**-4.158**	**<0.001**
	z Fruit Availability Index	C	0.048	0.037	1.289	0.198
	z Age of dependent offspring (y)	C	0.041	0.040	1.025	0.306
**Resting hours**	Intercept	I	-9.265	0.300	-	-
	Active time (h)	*Offset*				
	Site (Suaq vs. Tuanan)	C	1.604	0.338	-	-
$\chi^2_{8,16}$ = 23.85, P = 0.002, ΔAIC = 7.85	z Cumulative female association hours	F	-0.035	0.035	-1.023	0.306
	z Cumulative male association hours	F	0.042	0.072	-	-
	Consecutive days with female count	F	0.048	0.064	0.751	0.453
	**Consecutive days with male count**	F	-0.107	0.034	**-3.184**	**0.001**
	Number of copulations	F	-0.152	0.144	-1.058	0.290
	Male-female cumulative aggression index	F	0.213	0.126	1.689	0.091
	Female-female agonistic interactions (no vs. yes)	F	0.404	0.283	1.426	0.154
	**z Fruit Availability Index**	C	-0.137	0.064	**-2.148**	**0.032**
	**z Age of dependent offspring (y)**	C	-0.237	0.072	**-3.316**	**0.001**
	Social interaction time (h)	C	0.022	0.220	0.100	0.920
	**Site: z Cumulative male association hours**	F	0.177	0.085	**2.090**	**0.037**

Stable 12 LMM outputs of the full model for daily feeding, moving and resting hours of parous females (N=2086 full-day follows of 20 parous females and 279 follow periods) excluding daily active time as offset term. The comparison of each full model (reported model) to the control model is reported in the first column below the response variable. All fixed and control effects with P < 0.05 are indicated in bold. (*Note:* z = fixed effect variable was z-transformed prior to analysis; O = offset term; C = control factor, F = fixed effect)

Response	Fixed effects		Estimate	SE	t	P
**Feeding hours**	Intercept	I	6.772	0.256	-	-
	Site (Suaq vs. Tuanan)	C	-0.718	0.286	-	-
	z Cumulative female association hours	F	0.166	0.055	-	-
$\chi^2_{8,17} = 54.18$,	z Cumulative male association hours	F	-0.062	0.069	-	-
$P < 0.001$,	Consecutive days with female count	F	-0.100	0.062	-1.620	0.105
$\Delta AIC = 36.18$	**Consecutive days with male count**	F	0.069	0.033	**2.102**	**0.036**
	Number of copulations	F	-0.032	0.139	-0.231	0.817
	Male-female cumulative aggression index	F	-0.107	0.122	-0.875	0.381
	Female-female agonistic interactions (no vs. yes)	F	-0.447	0.275	-1.627	0.104
	z Fruit Availability Index	C	-0.003	0.058	-0.048	0.962
	z Age of dependent offspring (y)	C	0.096	0.064	1.495	0.136
	**Social interaction time (h)**	C	-1.014	0.213	**-4.758**	**<0.001**
	**Site : z Cumulative female association hours**	F	-0.260	0.062	**-4.179**	**<0.001**
	**Site : z Cumulative male association hours**	F	-0.214	0.081	**-2.643**	**0.008**
**Moving hours**	Intercept		1.960	0.090	-	-
	Site (Suaq vs. Tuanan)	F	-0.620	0.096	-	-
	z Cumulative female association hours	F	-0.018	0.025	-	-
$\chi^2_{8,17} = 63.39$,	**z Cumulative male association hours**	F	0.049	0.021	**2.348**	**0.019**
$P < 0.001$,	**Consecutive days with female count**	F	0.063	0.027	**2.281**	**0.023**
$\Delta AIC = 45.39$	**Consecutive days with male count**	F	0.031	0.015	**2.111**	**0.035**
	Number of copulations	F	0.014	0.081	0.170	0.865
	Male-female cumulative aggression index	F	-0.076	0.055	-1.380	0.168
	Female-female agonistic interactions (no vs. yes)	F	0.093	0.122	0.765	0.444
	**z Fruit Availability Index**	C	0.154	0.026	**5.962**	**<0.001**
	**z Age of dependent offspring (y)**	C	0.129	0.029	**4.556**	**<0.001**
	Social interaction time (h)	C	0.023	0.095	0.243	0.808
	**Site : z Cumulative female association hours**	F	0.080	0.027	**2.929**	**0.003**
	**Site : Copulation count**	F	0.197	0.097	**2.039**	**0.042**
**Resting hours**	Intercept	I	1.958	0.291	-	-
	Site (Suaq vs. Tuanan)		1.340	0.335	-	-
	z Cumulative female association hours		-0.053	0.052	-	-
$\chi^2_{8,17} = 64.85$,	z Cumulative male association hours		0.070	0.064	-	-
$P < 0.001$,	Consecutive days with female count	F	0.079	0.058	1.365	0.172
$\Delta AIC = 46.85$	Consecutive days with male count	F	-0.051	0.030	-1.683	0.093
	Number of copulations	F	0.060	0.130	0.466	0.641
	**Male-female cumulative aggression index**	F	0.230	0.114	**2.019**	**0.044**
	Female-female agonistic interactions (no vs. yes)	F	0.031	0.256	0.121	0.903
	z Fruit Availability Index	C	-0.054	0.050	-1.095	0.274
	**z Age of dependent offspring (y)**	C	-0.150	0.055	**-2.835**	**0.005**
	**Social interaction time (h)**	C	0.512	0.198	**2.586**	**0.010**
	**Site : z Cumulative female association hours**	F	0.146	0.058	**2.521**	**0.012**
	**Site : z Cumulative male association hours**	F	0.231	0.074	**3.113**	**0.002**

Stable 13 LMM output for activity budget changes of parous females at Tuanan in response to social, ecological and physiological factors (N = 1874 full day follows of 15 females and 250 FPs). Fixed factors with P < 0.05 are indicated in bold. Below the response variable the model comparison is reported based on likelihood ratio tests.

Reponse	Fixed Effects	Type	Estimate	SE	t	P
**Active hours**	Intercept		10.943	0.083		
	**z Cumulative female association hours**	Fixed	0.088	0.029	**3.046**	**0.002**
$\chi^2_{4,14} = 94.28$,	**z Cumulative male association hours**	Fixed	0.084	0.043	**1.951**	**0.051**
P < 0.01,	**Number of copulations**	Fixed	0.336	0.132	**2.551**	**0.011**
ΔAIC = 74.28	Male-female cumulative aggression index	Fixed	0.029	0.116	0.254	0.800
	Female-female agonistic interactions (no vs. yes)	Fixed	-0.318	0.213	-1.495	0.135
	**z Fruit Availability Index**	Control	0.144	0.043	**3.374**	**0.001**
	z Age of dependent offspring (y)	Control	0.085	0.047	1.807	0.072
	**Social interaction time (h)**	Control	0.593	0.171	**3.474**	**0.001**
	Consecutive days with female count	Fixed	0.054	0.051	1.064	0.287
	Consecutive days with male count	Fixed	0.037	0.029	1.282	0.200
**Feeding hours**	Intercept		-4.907	0.141		
	Active time (h)	Offset				
	**z Cumulative female association hours**	Fixed	-0.169	0.039	**-4.299**	**<0.001**
$\chi^2_{4,14} = 233.64$,	**z Cumulative male association hours**	Fixed	-0.337	0.058	**-5.823**	**<0.001**
P < 0.001,	**Number of copulations**	Fixed	-0.492	0.179	**-2.746**	**0.006**
ΔAIC = 213.64	Male-female cumulative aggression index	Fixed	-0.146	0.158	-0.929	0.353
	Female-female agonistic interactions (no vs. yes)	Fixed	-0.102	0.289	-0.352	0.725
	**z Fruit Availability Index**	Control	-0.146	0.056	**-2.592**	**0.010**
	z Age of dependent offspring (y)	Control	0.006	0.062	0.096	0.923
	**Social interaction time (h)**	Control	-1.651	0.232	**-7.110**	**<0.001**
	**Consecutive days with female count**	Fixed	-0.191	0.069	**-2.742**	**0.006**
	Consecutive days with male count	Fixed	0.017	0.039	0.444	0.657
**Moving hours**	Intercept		-9.614	0.095		
	Active time (h)	Offset				
$\chi^2_{4,7} = 18.66$,	z Fruit Availability Index	Fixed	0.014	0.041	0.333	0.740
P < 0.001,	z Age of dependent offspring (y)	Fixed	0.026	0.044	0.598	0.550
ΔAIC = 12.66	**Social interaction time (h)**	Fixed	-0.732	0.170	**-4.296**	**<0.001**
**Resting hours**	Intercept		-7.683	0.166		
	Active time (h)	Offset				
$\chi^2_{4,14} = 30.71$,	z Cumulative female association hours	Fixed	-0.012	0.041	-0.301	0.764
P < 0.001,	**z Cumulative male association hours**	Fixed	0.203	0.063	**3.238**	**0.001**
ΔAIC = 10.81	Number of copulations	Fixed	-0.114	0.189	-0.606	0.545
	Male-female cumulative aggression index	Fixed	0.210	0.166	1.270	0.204
	Female-female agonistic interactions (no vs. yes)	Fixed	0.385	0.302	1.273	0.203
	**z Fruit Availability Index**	Control	-0.194	0.070	**-2.780**	**0.006**
	**z Age of dependent offspring (y)**	Control	-0.225	0.078	**-2.899**	**0.004**
	Social interaction time (h)	Control	0.114	0.244	0.466	0.641
	Consecutive days with female count	Fixed	0.076	0.073	1.044	0.297
	**Consecutive days with male count**	Fixed	-0.085	0.041	**-2.065**	**0.039**

Stable 14 LMM output for activity budget changes of parous females in the Suaq population in response to social, ecological and physiological factors (N = 221 full day follows of 6 females and 30 FPs). Fixed factors with P < 0.05 are indicated in bold. Below the response variable the model comparison is reported based on likelihood ratio tests.

Reponse	Fixed Effects	Type	Estimate	SE	t	P
**Active hours**	Intercept		11.130	0.103		
	z Cumulative female association hours	Fixed	0.061	0.045	1.355	0.177
$\chi^2_{4,13}$ = 27.47,	z Cumulative male association hours	Fixed	0.034	0.052	0.648	0.518
P = 0.001,	Number of copulations	*(Omitted because of collinearity with male-female aggression)*				
ΔAIC = 9.47	Male-female cumulative aggression index	Fixed	0.049	0.104	0.473	0.636
	Female-female agonistic interactions (no vs. yes)	Fixed	-0.223	0.600	-0.372	0.710
	**z Fruit Availability Index**	Control	-0.216	0.070	**-3.103**	**0.004**
	z Age of dependent offspring (y)	Control	0.027	0.078	0.343	0.734
	Social interaction time (h)	Control	0.430	0.338	1.270	0.206
	Consecutive days with female count	Fixed	0.027	0.092	0.293	0.770
	Consecutive days with male count	Fixed	0.076	0.039	1.950	0.052
**Feeding hours**	Intercept		-4.740	0.434		
	Active time (h)	Offset				
$\chi^2_{5,14}$ = 19.97,	z Cumulative female association hours	Fixed	0.064	0.054	1.168	0.244
P = 0.02,	**z Cumulative male association hours**	Fixed	-0.137	0.065	**-2.105**	**0.036**
ΔAIC = 1.97	Number of copulations	Fixed	0.043	0.189	0.225	0.822
	Male-female cumulative aggression index	Fixed	-0.223	0.167	-1.334	0.184
	Female-female agonistic interactions (no vs. yes)	Fixed	0.269	0.727	0.370	0.712
	**z Fruit Availability Index**	Control	0.248	0.088	**2.812**	**0.009**
	z Age of dependent offspring (y)	Control	-0.110	0.111	-0.990	0.331
	**Social interaction time (h)**	Control	-0.948	0.420	**-2.257**	**0.025**
	Consecutive days with female count	Fixed	-0.014	0.112	-0.128	0.898
	Consecutive days with male count	Fixed	0.007	0.048	0.149	0.882
**Moving hours**	Intercept		-9.161	0.108		
	Active time (h)	Offset				
$\chi^2_{5,13}$ = 24.09,	z Cumulative female association hours	Fixed	-0.067	0.051	-1.320	0.188
P = 0.002,	z Cumulative male association hours	Fixed	0.036	0.058	0.624	0.533
ΔAIC = 8.09	Number of copulations	*(Omitted because of collinearity with male-female aggression)*				
	Male-female cumulative aggression index	Fixed	-0.160	0.117	-1.374	0.171
	Female-female agonistic interactions (no vs. yes)	Fixed	0.305	0.666	0.458	0.648
	**z Fruit Availability Index**	Control	0.222	0.063	**3.511**	**0.001**
	**z Age of dependent offspring (y)**	Control	0.156	0.074	**2.115**	**0.037**
	Social interaction time (h)	Control	-0.170	0.362	-0.469	0.640
	Consecutive days with female count	Fixed	-0.001	0.105	-0.011	0.991
	Consecutive days with male count	Fixed	-0.054	0.043	-1.259	0.209
**Resting hours**	Intercept		-9.178	0.209		
	Active time (h)	Offset				
$\chi^2_{5,14}$ = 16.88,	z Cumulative female association hours	Fixed	-0.089	0.059	-1.518	0.131
P = 0.05,	z Cumulative male association hours	Fixed	0.060	0.072	0.830	0.408
ΔAIC = 1.12	Number of copulations	Fixed	-0.119	0.210	-0.567	0.571
	Male-female cumulative aggression index	Fixed	0.228	0.179	1.278	0.203
	Female-female agonistic interactions (no vs. yes)	Fixed	0.131	0.798	0.164	0.870
	z Fruit Availability Index	Control	0.095	0.160	0.596	0.554
	z Age of dependent offspring (y)	Control	-0.315	0.183	-1.720	0.096
	Social interaction time (h)	Control	-0.457	0.485	-0.943	0.347
	Consecutive days with female count	Fixed	-0.052	0.121	-0.435	0.664
	**Consecutive days with male count**	Fixed	-0.140	0.054	**-2.577**	**0.011**

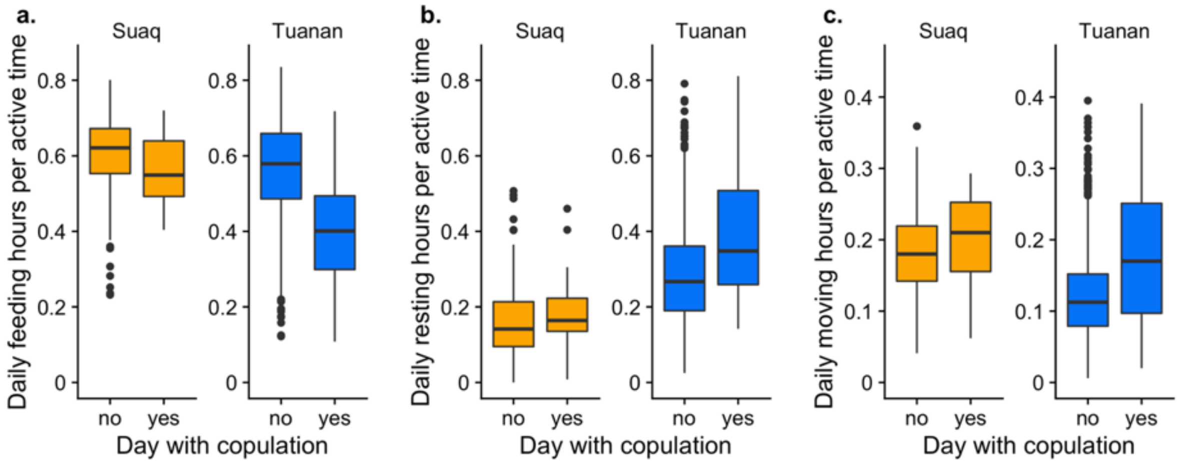

Sfigure 5 ***Daily feeding, resting and moving hour changes*** (per active time) (left to right) of parous females in relation to the occurrence of copulations (both forced and voluntary), by study site. The plot is based on the median of the raw data (female full-day focal follows).

FCM levels

The data set for the FCM levels was limited, especially for the high numbers of consecutive days in associations. Focal animals were normally followed for 5-10 consecutive days and the excretion of cortisol metabolites is delayed for 24-72 hours.

Stable 15 Output of the linear mixed model for the ln-transformed FCM levels (not standardized within individual and method) depending on social factors (consecutive days with males and females, and the occurrence of aggression), population, zFAI, the age of the dependent offspring, daily feeding proportions, the total days followed, the hour of sample collection, the days to sample extraction and the locality of hormone level analysis ($\chi^2_{5,17} = 21.67$, P = 0.04, ΔAIC=2.33; N=370 samples of 96 FPs, 21 IDs and 3 extraction methods). Fixed factors with P < 0.05 are indicated in bold. (*Note:* z = fixed effect variable was z-transformed prior to analysis; C = control factor, F = fixed effect)

	Type	**Estimate**	**SE**	**t**	**P**
Intercept		6.521	0.348		
Site (Suaq vs. Tuanan)	C	-0.468	0.351	-1.336	0.258
Consecutive days in association with female(s)	F	-0.042	0.051	-0.832	0.406
**Consecutive days in association with male(s)**	F	0.090	0.029	**3.082**	**0.002**
Male-female cumulative aggression index	F	-0.094	0.070	-1.345	0.180
Female-female agonistic interactions (no vs. yes)	F	-0.010	0.264	-0.037	0.971
Number of days followed	C	-0.003	0.010	-0.283	0.777
z Daily feeding proportion	C	-0.058	0.032	-1.790	0.074
z Age of dependent infant (y)	C	0.020	0.060	0.337	0.738
z Fruit Availability Index	C	0.036	0.053	0.676	0.502
Hour of sample collection	C	-0.002	0.015	-0.107	0.915
z Days to sample extraction	C	0.040	0.057	0.698	0.486
Laboratory (DPZ vs. IPB)	C	0.125	0.159	0.789	0.432

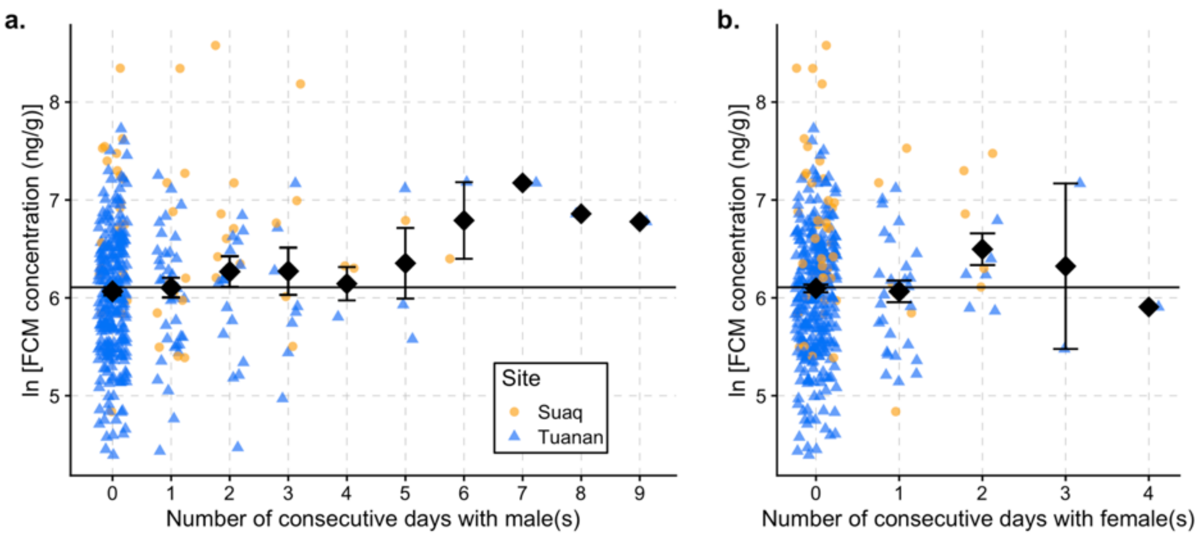

Sfigure 6 FCM levels (ln [FCM concentration (ng/g)]) (y-axis) of parous females in response to consecutive association days with males (a) and to consecutive association days with females (b). A jitter function was added to the plot to visualize the overlapping data points (consecutive days are only integers). *Note:* The black diamond shaped points indicate the mean FCM levels with the error bar. The black line indicates the overall mean of all available female FCM levels which were included in the analyses.

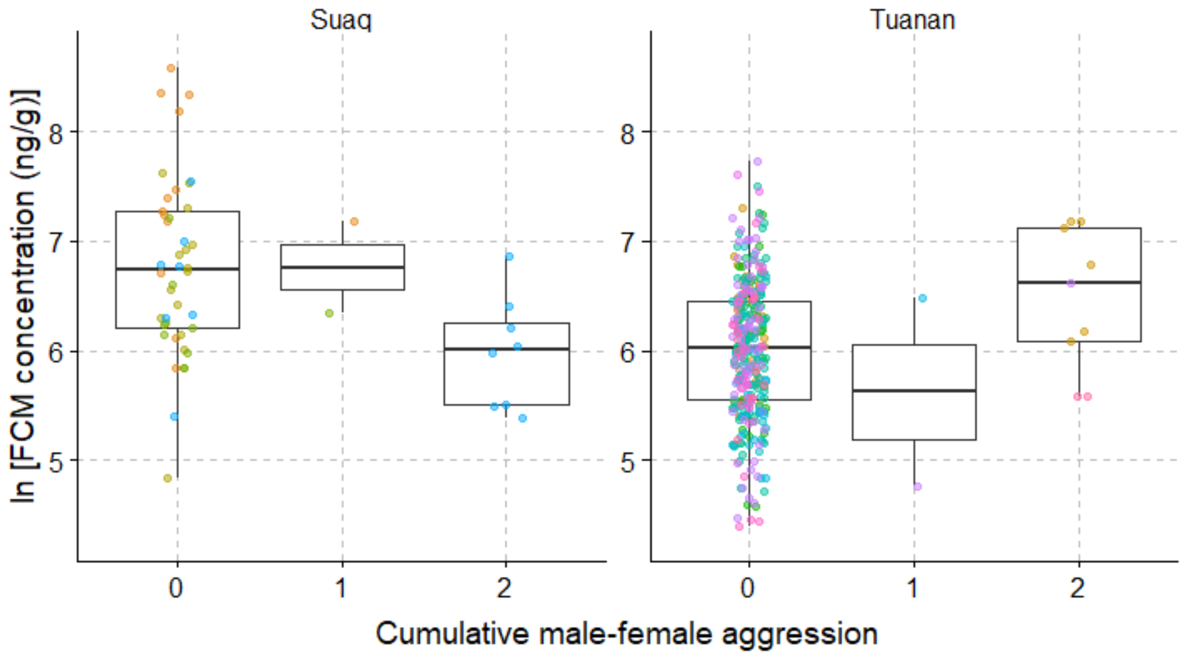

Sfigure 7 Female FCM levels (ln [FCM concentration (ng/g)]) in relation to cumulative male aggression (0 = no aggression, 1 = short, non-physical aggression (not in the direct sexual context), 2 = forced copulation or other form of sexual coercion) by study population. Different individuals are indicated in different color.
